# Hospital length of stay prediction for general surgery and total knee arthroplasty admissions: Systematic review and meta-analysis of published prediction models

**DOI:** 10.1177/20552076231177497

**Published:** 2023-05-29

**Authors:** Swapna Gokhale, David Taylor, Jaskirath Gill, Yanan Hu, Nikolajs Zeps, Vincent Lequertier, Helena Teede, Joanne Enticott

**Affiliations:** 1 22457 Faculty of Medicine, Nursing, and Health Sciences, Monash Centre for Health Research and Implementation, School of Public Health and Preventive Medicine, 2541 Monash University, Clayton, Victoria, Australia; 2 1890 Quality Planning and Innovation Unit, Eastern Health, Box Hill, Victoria, Australia; 3 Office of Research and Ethics, 1890 Eastern Health, Box Hill, Victoria, Australia; 4 5392 Department of Medicine, Alfred Health, Melbourne, Victoria, Australia; 5 Graduate Research Industry Partnerships (GRIP) Program, Monash Partners Academic Health Science Centre, Clayton, Victoria, Australia; 6 Eastern Health Clinical School, 2541 Monash University Faculty of Medicine, Nursing and Health Sciences, Box Hill, Australia; 7 Research on Healthcare Performance (RESHAPE), INSERM U1290, 27098 Université Claude Bernard Lyon 1, Villeurbanne, France; 8 Univ. Lyon, INSA Lyon, Univ Lyon 2, 133614 Université Claude Bernard Lyon 1, Lyon, France

**Keywords:** Risk, assessment, prediction, models, tools, factors, methods, length of stay, regression, machine learning

## Abstract

**Objective:**

Systematic review of length of stay (LOS) prediction models to assess the study methods (including prediction variables), study quality, and performance of predictive models (using area under receiver operating curve (AUROC)) for general surgery populations and total knee arthroplasty (TKA).

**Method:**

LOS prediction models published since 2010 were identified in five major research databases. The main outcomes were model performance metrics including AUROC, prediction variables, and level of validation. Risk of bias was assessed using the PROBAST checklist.

**Results:**

Five general surgery studies (15 models) and 10 TKA studies (24 models) were identified. All general surgery and 20 TKA models used statistical approaches; 4 TKA models used machine learning approaches. Risk scores, diagnosis, and procedure types were predominant predictors used. Risk of bias was ranked as moderate in 3/15 and high in 12/15 studies. Discrimination measures were reported in 14/15 and calibration measures in 3/15 studies, with only 4/39 externally validated models (3 general surgery and 1 TKA). Meta-analysis of externally validated models (3 general surgery) suggested the AUROC 95% prediction interval is excellent and ranges between 0.803 and 0.970.

**Conclusion:**

This is the first systematic review assessing quality of risk prediction models for prolonged LOS in general surgery and TKA groups. We showed that these risk prediction models were infrequently externally validated with poor study quality, typically related to poor reporting. Both machine learning and statistical modelling methods, plus the meta-analysis, showed acceptable to good predictive performance, which are encouraging. Moving forward, a focus on quality methods and external validation is needed before clinical application.

## Key messages

### What is known?

- Although classic regression and machine learning are commonly used in risk prediction of various patient outcomes like LOS in many specific and mixed disease groups, there is an urgent need for external validation and impact assessment studies in the generalized risk prediction literature.

### What does this study add?

- To our knowledge, this is the first systematic review assessing quality of risk prediction models for hospital LOS in general surgery and TKA groups using the PROBAST tool. Most of the studies showed a high risk of bias, attributable primarily to poor reporting of analysis and evaluation of model performance. There is wide variation in the numbers and types of predictor variables, data pre-processing, model development processes, and model evaluation metrics. We propose a list of suggested predictor categories for inclusion in predictive models for these groups.

### How might this study affect research, practice, or policy?

- This review adds evidence to the global imperative for improved reporting of prediction models and need for external validation of existing models; both of which are required to advance the field and move closer towards delivering feasible data-driven solutions that can impact patient care and better resource management.

## Introduction

Most Organization for Economic Co-operation and Development (OECD) countries have seen an increase in health spending with the latest estimates showing a 5% growth in 2020, and hospital inpatient and outpatient services make up the bulk of the health spending for all the OECD countries. Australian health expenditure has increased by an average of 2.7% per year in the last 18–20 years, and the cost of hospital care accounted for 40% of the total, of which 61.7% was spent on acute admitted care.^1,2^ Length of stay (LOS) in an acute hospital is a significant influencer of the cost of delivering hospital-based care and is a key measure of hospital performance (efficiency) according to the Australian Health Performance Framework.^3^ Extended LOS increases the risk of hospital-acquired infections and impacts patient flow and access to healthcare.^4^ According to a recent report, there is up to three- to four-fold variation in the average LOS in Australian hospitals^2^ which is due to a complex interaction of multiple factors, including some unrelated to the patient’s condition. Reducing unwanted variation in LOS is essential in Australia and globally to ensure the sustainability of economically viable health services for the future.

To utilize healthcare resources efficiently, studies have been undertaken globally that use existing data and apply statistical techniques like machine learning (ML), to create and validate predictive models for early identification of patients at risk of an extended LOS.^5–9^ Accurate risk prediction can enable targeted interventions to streamline care, reduce unnecessary LOS, and potentially impact system-level management of patient flow issues by providing high-level visibility of impending access issues and enabling proactive decision-making.^1,10^ Prior studies have investigated LOS prediction in specific disease groups like heart failure,^11^ cardiac surgery,^12^ thermal burns,^13^ or hospital populations like intensive care unit (ICU) and neonatal care.^14,15^ Other recent reviews have looked at this outcome from a risk adjustment perspective^16^ or a broad epidemiological perspective.^17^ Lequertier et al.^17^ examined literature published until September 2019 using three databases and reported common factors/features. They reported that over the last decade, ML is increasingly being used for complex problem solving using big data.

We aimed to extend the Lequertier review by broadening the search, evaluating risk of bias^18^ of the included studies, and adding data from recent 2 years’ to capture the emerging artificial intelligence (AI)/ML approaches. This review aims to assemble evidence from the international literature on optimal tools that identify patients at risk of an extended LOS and to assess the study methods (including prediction variables), study quality, and performance of predictive models (using area under receiver operating curve (AUROC)) for general surgery and total knee arthroplasty (TKA) populations. There have been multiple studies reporting procedure-specific outcome prediction models; however, considering the formative stages of many healthcare information systems, it may be challenging to deploy multiple discrete predictive models to predict LOS in specific populations. Having a generalized model that can be applied to a larger population with procedure-specific considerations built in represents greater feasibility. Recent advances in TKA have seen a significant decline in the post-operative LOS in these populations.^19,20^ We considered this procedure-specific group as a case study of interest as it represents a population with potential to reduce variability and improve efficiency. This updated evidence can then assist efforts to advance the field and move closer towards delivering feasible data-driven solutions that can impact patient care and better resource management.

## Methods

‘Prediction tools’ or ‘tools’ for this review can include any type of risk assessment tools/flags/factors or risk prediction models that used computerized statistical methods for predicting hospital LOS. This review was conducted according to the Preferred Reporting Items for Systematic Reviews and Meta-Analyses (PRISMA) guidelines.^21^ Protocol was registered on the International Prospective Register of Systematic Reviews (PROSPERO) (https://www.crd.york.ac.uk/PROSPERO/) (#CRD42021272198).

### Search strategy

We searched CINAHL, EMBASE, OVID MEDLINE, OVID EMCARE, and Cochrane databases systematically on 31 August 2021 using a predefined search strategy guided by our library scientist (VD) available in Supplementary Table S2. Primary concepts searched were ‘risk factors’, ‘statistical/prediction models’, and ‘length of stay’. Considering the rapidly advancing field of health data analytics, we narrowed the search to only include English language articles, from OECD comparable countries and published after 2010.

There have been significant advances in the field of health data analytics methodologies in the last 10 years with frequent use of ML methods.^22,23^ Taking into consideration advancing patient age, comorbidities and complexity, rising hospital bed pressures, and shorter stays as well as evolving methods and standards for prediction modelling, we restricted our review to studies published after 2010.

Reference lists of included publications were examined to identify any additional potential studies. Grey literature search using key terms was completed in Google and Google Scholar in a time-limited way (20 hours over 4 weeks).

### Eligibility criteria

As shown in Supplementary Table S3, we included primary studies that reported LOS predictive tools for adults admitted to acute care hospitals that reported prediction metrics^24^ to inform what works in LOS prediction methods and in what context. No limits on publication types were applied. We excluded studies looking at day procedures (LOS < 24 hours) and those describing or including admissions to nursing homes, or community hospitals/rehabilitation facilities due the difference in their operational structure and purpose, compared to the acute hospital setting.

LOS for general surgery was the clinical area of focus. As a separate case study, we also examined TKA. TKA was chosen after examining reports from Australian Institute of Health and Welfare (AIHW) to explore diagnoses with high relative LOS.^1–3^ TKA is a common high-volume surgery group with evidence-based protocols, and the variability in TKA procedures is expected to be less than general surgery procedures.

Studies that were not primary research were excluded, including conference abstracts, unpublished studies, book chapters, and review articles. We also excluded reports focussing on single diagnosis or specialized surgical groups (apart from TKA), including obstetric, paediatric, cardiac, and cancer surgery and studies that did not assess LOS as an outcome.

### Study screening and data extraction

Screening, full-text review, data extraction, and quality assessment were completed using the web-based data management platform Covidence^25^ and EndNote X9.3.3 (Clairvate). Title, abstract, and full-text screening were conducted by two reviewers (SG/JG), who were responsible for selecting studies for inclusion. In case of discrepancies, consensus was reached via discussion. SG extracted data based on CHARMS and TRIPOD^26,27^ into a predefined data extraction table (Supplementary Table S4).

### Quality assessment

Risk of bias was assessed independently by two reviewers (SG/YH) based on PROBAST recommendations. Disagreement was resolved with a third reviewer (JE), and consensus reached. Using PROBAST,^28^ studies are rated as low, moderate, and high concern for bias and applicability in each of four domains—participants, predictors, outcomes, and analysis.^18,27^ We used guidance from the adaptation of the PROBAST tool for ML models reported elsewhere.^29^

### Data synthesis

The extracted information from the included articles is provided in Supplementary Table S5. Data sources used to create and/or validate the LOS prediction model were classified as (a) administrative/registry/claims and (b) medical records and prediction modelling methods as classical statistical methods (regression models)/ML/both.

Model performance measures of discrimination and calibration were extracted and synthesized. Discrimination measures, where possible, were presented as AUROC with 95% confidence intervals (CIs).^21^ We applied an AUROC of 0.5 to suggest no discrimination (ability to diagnose patients with and without the risk under test), 0.7 to 0.8 acceptable discrimination, 0.8 to 0.9 excellent discrimination, and more than 0.9 as outstanding.^30^ Calibration was assessed from any visual calibration plots reported, where available, or using calibration statistics.^30,31^

Predictor variables in the included LOS models were classified into categories adapted from the recent systematic review by Lequertier et al.^17^ as shown in Table 1. Level of validation (development with or without internal validation and/or external validation) was based on PROBAST guideline.^28^

**Table 1.** Predictor variable categories with possible inclusions.

	Predictor variable category	Example inclusions
1.	Administrative	Type of insurance, Workcover/TAC, etc.
2.	Demographic and anthropometric	Age, sex, BMI, ethnicity, carer status/living situation
3.	Diagnosis (primary and secondary including comorbidities) and procedure types	Diabetes, hypertension, COPD, ICD diagnostic codes, etc.
4.	Physical examination (biological and physiological parameters)	Serum haemoglobin, albumin, globulin, BSL, etc.
5.	Risk scores	Frailty Index, Charlson Comorbidity Index (CCI), American Surgical Association (ASA) Score
6.	Admission characteristics	Emergency/elective, from home/residential care
7.	Hospital characteristics	Rural vs urban, teaching, private/public, etc.
8.	Healthcare professional characteristics	Years of experience, volume of surgery, seniority
9.	Documentation and clinical notes	Structured and unstructured text from medical records
10.	Medications	Psychotropic drugs, insulin, opioid medications, etc.

TAC: Transport Accident Commission.

### Meta-analysis

The most common performance measure reported (AUROC) for the general surgery LOS models was examined with random-effects meta-analysis using restricted maximum likelihood estimation. Random effects meta-analysis allows for heterogeneity across included studies. An estimate of the mean of the underlying random-effects distribution is routinely reported to summarize the meta-analysis. An accompanying Confidence interval (CI) is also regularly presented to show the precision of the effect estimate.^32^ CIs represent a range of values that are likely to contain the true mean value of some response variable based on specific values of one or more predictor variable.^33^ Recently, there is a growing body of literature recommending a need for summary estimate that comprehensively accounts for the within-study differences. The prediction interval which shows the range of effect estimates in future studies has been proposed to be presented in meta-analyses.^34–36^ As prediction intervals attempt to create an interval for a specific new observation, there's more uncertainty in our estimate and thus prediction intervals are always wider than CIs.^33^

Stata SE 17 was used for statistical analysis and calculation of 95% prediction intervals, to provide a point estimate for the estimated performance of the model in a new population. When standard error of AUROC was unreported, it was estimated using methods by Hanley and McNeil^37^ and Kottas et al.^38^ Heterogeneity was reported as *I*^2^.^39^ The number of eligible validation studies was small; hence, further investigation of sources of heterogeneity was not possible.

#### Publication bias

Forest plots showing effect sizes and CIs were generated. Egger’s regression was used for evaluating funnel plot asymmetry due to small study effects.^31,40^

## Results

**Figure 1.**
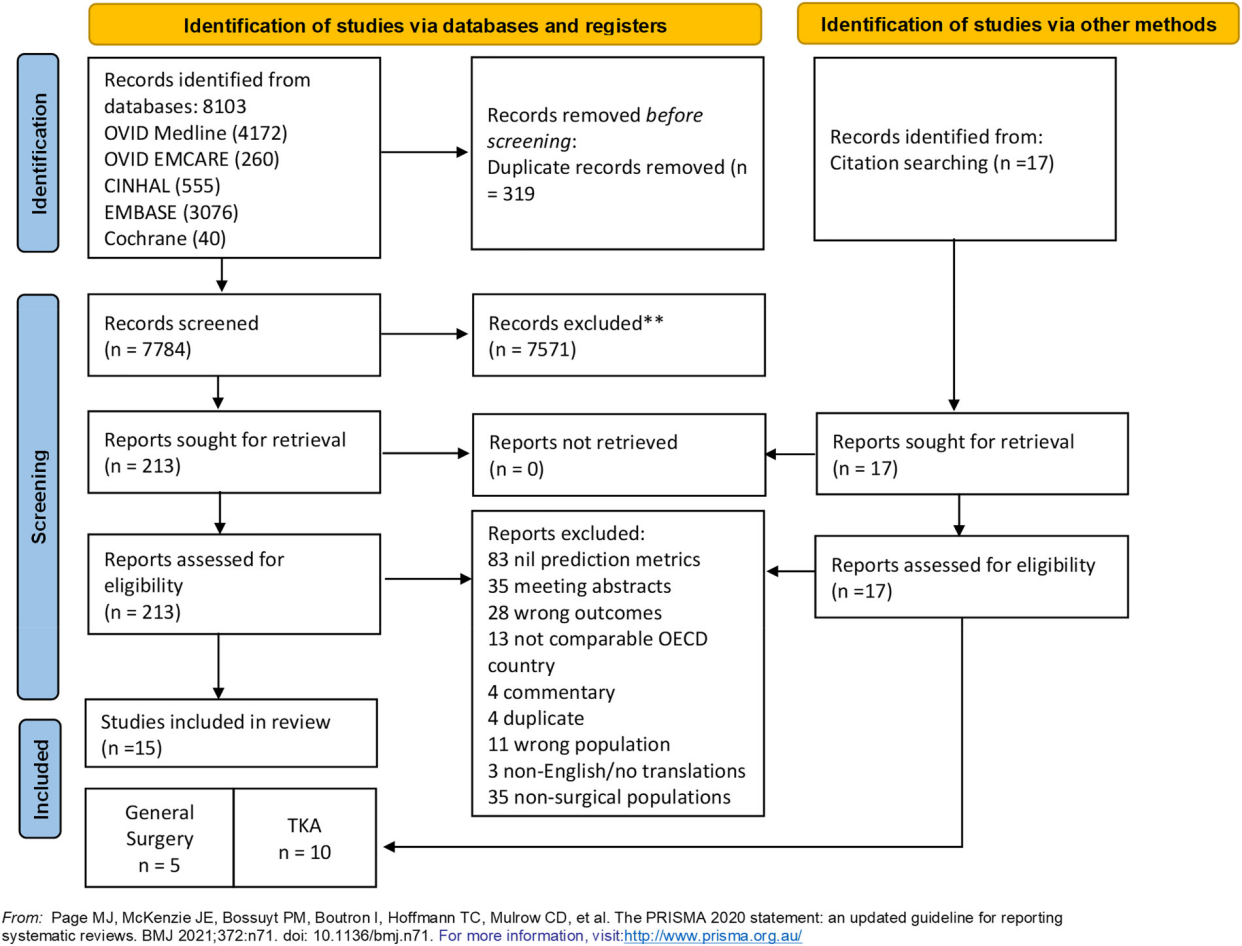
PRISMA flow diagram. This chart demonstrates the systematic review of the literature for hospital length of stay prediction tools. PRISMA: Preferred Reporting Items for Systematic Reviews and Meta-Analysis; TKA: total knee arthroplasty.

The search yielded 8103 studies from OVID MEDLINE (4172), OVID EMCARE (260), CINHAL (555), EMBASE (3076), and Cochrane.^40^ Records were exported to Covidence, 319 duplicates removed, and the remaining 7784 records were screened, which yielded 213 potential reports for full-text retrieval. Citation searching identified an additional 17 records which were assessed for eligibility. Following full-text review, 15 were selected for inclusion based on the eligibility criteria: 5 reporting on general surgery populations and 10 on TKA. PRISMA diagram illustrates the search in Figure 1. Study characteristics are summarized in Supplementary Table S5.

### General surgery prediction models

Of the five general surgery prediction model studies included in this review, two were published since 2019; two were from the USA and Canada each and one from Europe. All the studies were observational with three prospective and two retrospective studies. Detailed study characteristics are summarized in Supplementary Table S5.

In the 5 included studies, there were 15 models (multiple models reported in each study). The median study duration was 4 (range 0.7–5) years with a median sample size of 1165 (range 300–35,179,507). Prolonged LOS cut-off threshold varied from 5 to 14 days. Of the 15 models reported, 3 were external validation studies^41,42^ of the ‘Risk Stratification Index’ (RSI) tool.^42^

#### Data sources

Three of the 5 studies (7/15 models) reported using administrative data, and 2 (8/15 models) used medical records. All LOS was predicted post discharge, with 3 studies demonstrating some capability for prognostic prediction on admission. LOS was modelled as a categorical/binary variable in 4 and continuous in 1 study.

#### Predictive modelling methods

The general surgery group’s validation level was good with three externally validated studies. Of the 15 general surgery models (multiple models reported in each study), all models used classical statistical approaches: Cox regression (*n* = 5), multivariable logistic/linear regression (*n* = 6), and classification analysis (*n* = 4).

#### Analytical pipeline

The median number of predictors/variables was 12 (range 7–1094), with all models including all candidate predictors in multivariable modelling. Within the 15 included models, the model selection method was poorly reported (not specified in 6, stepwise forward/backward selection in 5, and full model approach in 4). Handling of missing data was not reported in 7/15 models, and patients with missing data were excluded in the remaining 8/15 models. Methods used to manage overfitting and optimism reported in 9 models included bootstrapping and sensitivity analysis.

#### Reported performance metrics and interpretation

There was a wide range of predictive metrics used for reporting of model performance. Frequency of the reported performance measures (predictive metrics) is summarized in Supplementary Figure S2 and Table S6. Table 2 shows detailed information on the predictive models used in these studies.

**Table 2.** General surgery LOS prediction models included in systematic review (*n* = 15).

Study	Type of final model (Dev/Val)	Input variables	Variable selection methods	Outcome	Name of data analysis/modelling method used DD	AUROC values (95% CI)	Other prediction metrics
General surgery							
McIsaac 2020 (1)^43^	Internal validation	Base model: age, sex, ASA score, procedural risk	All candidate predictors	LOS > 6 days	Multivariable logistic regression	0.73	
McIsaac 2020 (2)		Base model + Clinical Frailty Scale^44^		LOS > 6 days		0.76	
McIsaac 2020 (3)		Base model + Fried phenotype^45^		LOS > 6 days		0.74	
McIsaac 2020 (4)		Base model + Frailty Index^46^		LOS > 6 days		0.74	
Almeida 2013	Development	NRS2002 low vs high risk	All candidate predictors	LOS > 10	Classification analysis		Sensitivity/specificity: 88 (85–92)/45 (35–42)
		MUST low vs medium + high risk					88 (85–92)/45 (35–42)
		SGA (nil vs moderate/severe)					85 (79–87)/93 (87–95)
		Recent weight loss ≥5%					89 (86–92)/71 (69–74)
Sigakis 2013^41^	External validation	ICD Diagnosis and Procedure codes used in Risk Stratification Index model	Stepwise hierarchical selection	Median LOS for population	Cox proportional hazards	0.884 (0.882–0.886)	
Sessler 2010 (1)^42^: all admissions	Internal validation	ICD Diagnosis and Procedure codes used in Risk Stratification Index model	Stepwise hierarchical selection	Median LOS = 5	Cox regression	0.865 (0.865 0.865)	
Sessler 2010 (2): surgical admissions				Median LOS = 5		0.896 (0.896 0.897)	
Sessler 2010 (3)	External validation			Median LOS = 3		0.886 (0.883–0.888)	
Sessler 2010 (4)	External validation	ICD Diagnosis and Procedure codes used in Risk Stratification Index model + demographics: age, sex, and race	Stepwise hierarchical selection	Median LOS = 3		0.887 (0.885 0.889)	
Sutherland 2019^47^	Internal Validation	Model 1: case mix, age, gender, and comorbidity level	All candidate predictors	Median LOS = 4	Multivariable linear regression		Adjusted *R*^2 ^= 0.337
		Model 2: model 1 + PROM	All candidate predictors				Adjusted *R*^2 ^= 0.372

ASA: American Society of Anaesthesiologists; NRS 2002: Nutritional Risk Score 2002; MUST: Malnutrition Universal Screening Tool; SGA: Subjective Global Assessment; ICD: International Classification of Disease; PROM: patient-reported outcome measures.

#### Discrimination

AUROC/*C*-statistic was most frequently reported (9/15 models). The median values of AUROC were 0.75 (range 0.73–0.886), indicating fair to good discriminative ability in majority of models.^43^

#### Calibration

Calibration metrics were reported in only 4/15 general surgery models (likelihood ratio tests and calibration plots), and in these, the models appeared to be sufficiently calibrated.

Of the 5/15 general surgery models (2/5 studies) reporting comprehensive performance metrics, including calibration, discrimination, and overall accuracy measures, a model using various frailty risk scores by McIsaac et al.^43^ demonstrated an acceptable level of discriminative ability with AUROC of 0.76 and good calibration. Sigakis et al.^41^ reported higher discrimination on internal (AUROC 0.884 (0.882–0.886)) and external validation (AUROC 0.886 (0.883–0.888)) using the Risk Stratification Indices as predictors. Both studies had a moderate risk of bias due to poor reporting of overfitting and excluding >5% sample for missingness, respectively.^48^

#### Predictors/variables

Variable importance in the studies was reported using association metrics like hazard ratio, incident rate ratio, and estimates/regression coefficients. The most frequently used predictors are outlined in Table 3. Comorbid diagnostic groups (mental health diagnoses^42^ and depressive disorders^47^) and case mix categories like lower gastrointestinal surgeries^43,47^ emerged as influencers of risk of prolonged LOS in 11/15 models (4/5 studies); however, 2/3 studies were evaluating the RSI derived from risk associated with individual diagnostic and procedure codes. Various risk assessment tools like nutritional risk screening,^49^ frailty risk,^43^ comorbidity, and presurgical risk assessments^43,47^ were also used in 10/15 models (3/5 studies). Age,^43,47^ sex, and procedural risk were other variables included.^43^ Due to the small size of this group, robust conclusions about variable frequency were limited. However, it was clear that LOS predictions in this group were largely driven by the specific procedure type combined with comorbidities assessed using either screening tools or diagnostic codes.

**Table 3.** Frequently included input variables (predictors) in risk prediction of prolonged LOS in general surgery models (*n* = 5).

Input variables (predictors)	Frequency of inclusion in LOS risk prediction studies (*n* = 5)
**Diagnoses and procedure types (primary/secondary including comorbidities) **Alcohol rehabilitation and detoxification, Bipolar I disorder, most recent episode (or current) mixed Bipolar I disorder, most recent episode (or current) manic, Case mix category 221—colostomy/enterostomy, Case mix category 223—open large intestine/rectum resection without colostomy, planned Case mix category 226—non-major excision/repair of upper gastrointestinal tract, planned Case mix category 227—endoscopic large intestine/rectum resection without colostomy, Diagnostic related groups like Other electroshock therapy, Other psychiatric drug therapy, Paranoid type schizophrenia, chronic with acute exacerbation, Procedural risk.	4
**Risk scores** (CFI/ASA) ASA score Comorbidity levels 1–4, Frailty Assessment tools like Frailty Index (FI), Clinical Frailty Score (CFS), and Fried’s phenotype (FP), Nutritional risk measures like MUST, NRS2002, recent weight loss ≥5%, and SGA (nil vs moderate/severe), Patient-reported outcome measures (PROM)–PEG pain scores and PROM-PHQ-9 depression	3
**Demographic and anthropometric** Age and sex: female	2

#### Quality assessment

Quality assessment results are outlined in Table 4. The TRIPOD reporting guidelines for prediction model studies were published in 2015 and the PROBAST guidance for quality assessment of prediction model studies in 2019. Understandably, the three studies^41,42,49^ in our cohort that were published prior to 2015 cannot be strongly criticized for lack of reporting rigour especially around the analysis. Although many retrospective studies were done using secondary data sources, the majority were deemed to be from high-quality databases with acceptable reporting standards. Overall level of validation was high with two external validation studies including three discrete models. Participant and outcome risk of bias and applicability concern were noted to be low in four of five studies and high in one study. Risk of Bias (ROB) from predictor measurement was low in all the included studies. ROB for analysis and overall ROB were moderate in three of five studies and high in two of five studies. The commonly observed pitfalls were lack of comprehensive reporting of model performance measures (no calibration measures), reporting of missing data,^48^ and handling of data complexity. The overall applicability concern was low in three of five studies suggesting that results could be translated to our target population of general surgery admissions.

**Table 4.** Risk of bias assessment of general surgery (*n* = 5) using PROBAST tool.

	Type of prediction model	Participant risk of bias	Participant applicability	Predictor risk of bias	Predictor applicability	Outcome risk of bias	Outcome applicability	Analysis risk of bias	Overall risk of bias	Overall applicability concern
Almeida 2013	Development only	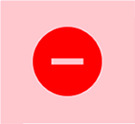	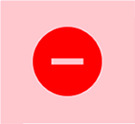			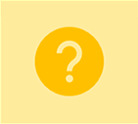	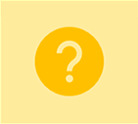	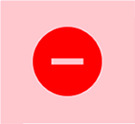	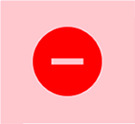	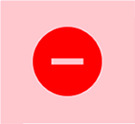
McIsaac 2020	Development only							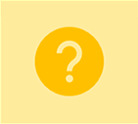	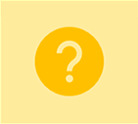	
Sigakis 2013	Validation only							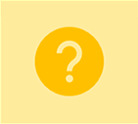	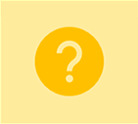	
Sessler 2010	Development and validation							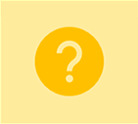	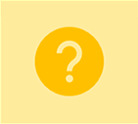	
Sutherland 2019	Development only							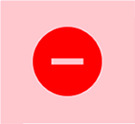	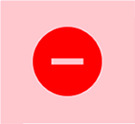	

### TKA case study

Of the 10 TKA studies included in this review, 5 were published since 2019; 4/10 were from the USA, 2 each from Europe, the UK, and Canada. All studies were observational, with an equal number of prospective and retrospective studies (*n* = 5). Median study duration was 2.9 (range 1.6–7) years with a median sample size 4466.5 (range 155–171,025). Administrative data sources were more common as compared to medical records. Majority of the studies collected data at preadmission/admission (9/10), with 2 studies complementing this with post-discharge or perioperative data. The cut-off for defining prolonged LOS ranged from 1 to 5 days. LOS and was predicted categorically in 8/10 studies and continuously in 2. The level of validation was lower compared to the general surgery models, with only one study showing external validation of their model. There were 22 models reported in the 10 studies. Although classical statistical approaches were more common (18/22), 2 studies (4 models) used Bayesian modelling^50^ and deep learning approaches^51^ for prediction.

The median number of predictors/variables used in TKA studies was 9 (range 2–20), with most studies including all candidate predictors in multivariable modelling. Commonly noted analytical issues were pre-selection of variables based on univariable analysis and poor reporting of handling of missing data (6/10 studies). Reported methods random split and cross-validation were used to manage overfitting and optimism in five studies while the rest did not report these. Poor reporting of prediction model parameters, lack of comprehensive model performance measures, and lower validation levels meant that 9/10 studies in this group were at a high ROB. Noteworthy is that only three of the nine studies were published in or prior to 2015 when the TRIPOD guidelines were released.

#### Predictor variables

Demographic variables, like age > 75, sex, living situation (alone) or lack of carer, socioeconomic factors, ethnicity, body mass index (BMI), and smoking status, and risk scores, like risk of mortality, severity of illness, Charlson Comorbidity Index, and mobility scores, were used consistently in 9/10 studies (Table 5). Diagnoses like diabetes, hypertension, and cardiovascular disease and procedural factors like implant type, operative joint complexity, surgery start time p.m., and surgical technique were also widely included in 8/10 studies. Admission characteristics (e.g. day of admission (Friday to Sunday), discharge destination, and type of admission) were included in over half of the models (Table 5).

**Table 5.** Frequently included input variables (predictors) in risk prediction of prolonged LOS in TKA models (*n* = 10).

Input variables (predictors)	Frequency of inclusion in LOS risk prediction studies (*n* = 10)
Demographic and anthropometric Age ≥75, sex, BMI, living situation (home/not home), income quartile/social deprivation, education, and ethnicity/race (white vs other)	9
Diagnoses (primary/secondary including comorbidities) and procedure types Comorbid diagnoses like diabetes (IDDM/NIDDM), hypertension, history, or number of cardiac/renal/pulmonary conditions Pharmacologically treated psychiatric disorder Previous stroke or transient ischaemic attack (TIA) Other musculoskeletal diagnoses Current smoking status Procedural factors like implant type, operative joint hip vs knee, replacement type, surgery start time p.m., surgery type (TKA vs UKA), surgical technique, and surgical volume >10	8
Risk scores Comorbidity risk scoring tools like APR risk of mortality, APR severity of illness, ASA scores 3–4, BRASS, Charlson comorbidity score (CCI), functional assessments like KOOS, Veterans Rand-12 (VR-12) score, and use of walking aid	9
Admission characteristics Day of admission (weekend/weekday), expected discharge destination: usual residence, transferred from outside hospital, and type of admission (emergency vs planned)	5
Physical examination (biological and physiological parameters) Serum levels of albumin, creatinine, platelets, white blood cell (WBC) count, and glycosylated haemoglobin HbA1c (≥7%) Anaemia	3
Hospital characteristics Hospital site or type (rural/urban teaching/urban non-teaching)	2
Medications Use of anticoagulants	1
Healthcare professional characteristics Consultant	1

### Meta-analysis

We conducted meta-analysis on the three general surgery validation models that used the RSI tool to predict LOS above or below the median using a time to event Cox proportional hazards regression analysis. The meta-analysis reports the 95% prediction interval to account for varying model performance due to differences in case mix and other study level factors.^52^

**Figure 2.**
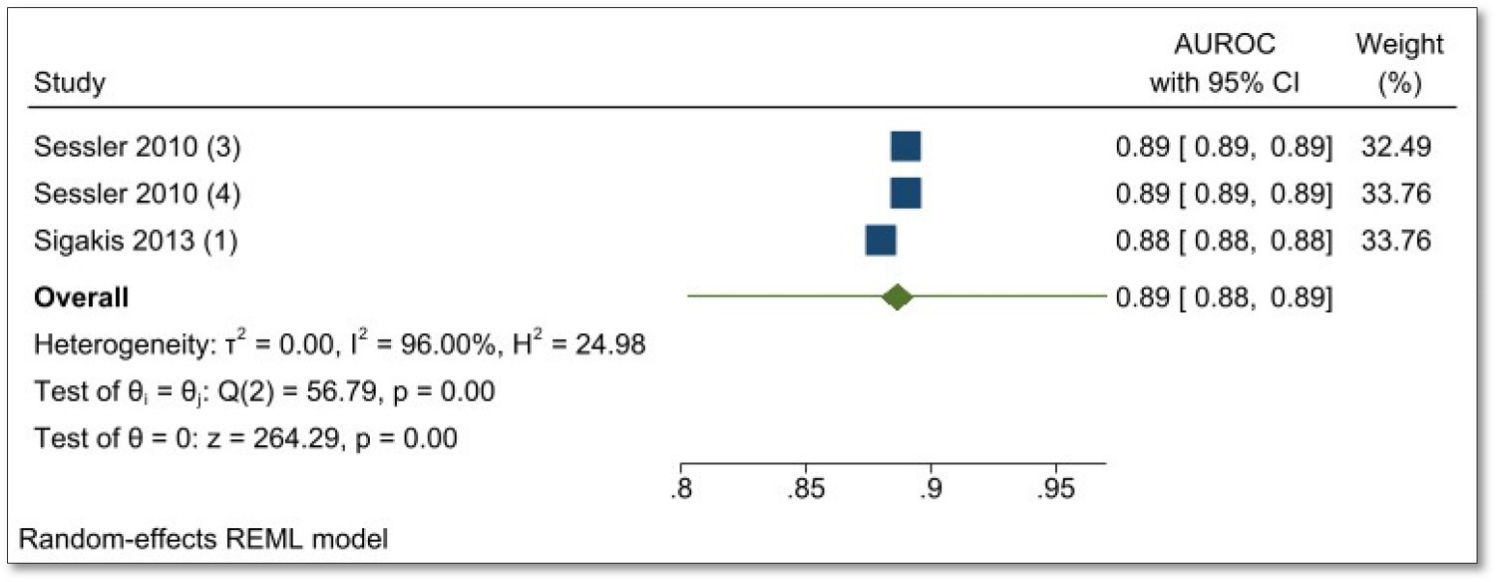
Forest plot of meta-analysis of validation studies in general surgery (*n* = 3).

Meta-analysis for AUROC values of validation in general surgery studies (shown in Figure 2 (forest plot)) shows 95% prediction interval for theta of 0.803 and 0.970 (*I*^2 ^= 96%, Table 6).

**Table 6.** Meta-analysis summary for three validation studies in general surgery.

Number of studies = 3	
Random-effects model	Heterogeneity
Method: REML	tau^2^ = 0.0000
	*I*^2^ (%) = 96.00
	*H*^2^ = 24.98
95% prediction interval for theta = (0.803, 0.970)	
Test of theta = 0: *z* = 264.29; Prob > |*z*| = 0.0000	
Test of homogeneity: *Q* = *χ*^2^(2) = 56.79; Prob > *Q* = 0.0000	

Sources of heterogeneity were not explored further statistically due to the small sample size. However, Table 7 outlines the differences in study populations and characteristics. The included validation studies did not report individual participant data (IPD) to enable calculation of the calibration metrics; however, the models reportedly showed poor calibration overall.

**Table 7.** Study and population characteristics of included validation studies in meta-analysis (*n* = 3).

Study	Total sample size	Predictors	Age	Median LOS for population	Risk stratification index		AUROC values (95% CI)	Calibration metric
					Diagnosis code count	Procedure code count		
Sigakis 2013	108,423	Risk Stratification Index model	54.5 ± 19.9	6.6 (0–16.2)	6.9 ± 2.9	3.0 ± 2.4	0.884 (0.882–0.886)	Calibration plot, nil slope/intercept
Sessler 2010 (3)	101,202	Risk Stratification Index model	56.6 ± 16.0	3 [1,6]	6.8 ± 2.9	3.3 ± 2.3	0.886 (0.883–0.888)	Calibration plot, nil slope/intercept
Sessler 2010 (4)	101,202	Risk Stratification Index model + demographics: age, sex, and race	56.6 ± 16.0	3 [1,6]	6.8 ± 2.9	3.3 ± 2.3	0.887 (0.885 0.889)	Calibration plot, nil slope/intercept

#### Publication bias

Funnel plot (Supplementary Figure S3) and Egger's test (Supplementary Table S9) did not reveal any publication bias or small study effects in our cohort of validation studies.

## Discussion

This systematic review of risk prediction models for prolonged LOS for general surgery showed only classical statistical models used for general surgery LOS predictions, and meta-analysis of the three externally validated general surgery model discrimination (AUROC) performance values indicated excellent predictive performance. However, model calibration, needed for impact assessment on implementation, was not consistently reported, resulting in poor study quality, typically related to poor reporting and lack of adoption of existing guidelines. We also identified prediction models for prolonged LOS for TKA as a case study of a common surgery, showing a sharp rise in studies occurring in the past 2 years. Both ML and classical statistical methods were employed and showed acceptable to good discriminative ability, suggesting that both approaches can predict LOS. Again, model calibration, needed for impact assessment on implementation, was not consistently reported, resulting in poor study quality rankings.

Only three external validation model performance outputs could be included in the meta-analysis while maintaining consistency of prediction variables included in the models. The 95% prediction intervals suggested that the predictive performance of the model on application to an independent population is likely to fall in the range of 0.803–0.970, which implies excellent discriminatory ability for general surgery LOS. These results must be interpreted with caution due to small sample size and limited generalizability to wider hospital populations. In hospital settings, LOS predictions have a dual benefit in being a proxy measure of clinical outcomes as well as hospital efficiency.^53^ Literature about procedure-specific prediction models with good prediction accuracy^54,55^ is abundant with models primarily predicting clinical outcomes like 30-day mortality and post-operative pain. As such, population-based LOS predictions are key enablers of organizational resource planning as well as the daily access and flow issues managed by the frontline staff. Recent study by Xu and colleagues^56^ has shown good LOS predictive capability in all-inclusive elective surgery population. Hence, the purpose of prediction should guide the choice of procedure-specific vs population-specific models. Heterogeneous LOS models that provide accurate predictions could enable service planning for efficient scheduling and patient identification for perioperative optimization for surgical patients and offer a single system of risk prediction at the point of care, presenting an opportunity to intervene ‘just-in-time’ for unplanned admissions. Deep learning techniques that account for population heterogeneity might be the way forward if demonstrating acceptable model performance (including large sample size and ensemble methods).^57^

Overall, prediction models for LOS in general surgery and TKA populations were infrequently externally validated. Similar observations about a lack of external validation studies have been noted globally for most areas of clinical risk prediction, thereby limiting generalizability.^17,58–60^ Contributing factors related to the inconsistency in the predictor variables used in the various models have been proposed, which may indeed be true for our review as the number of variables ranged from 2 to 1096 in the LOS models included. Contrariwise, a recent meta-analysis on gestational diabetes mellitus risk prediction revealed that the same six to eight predictor variables were included in most models including external validation studies.^61^

Systemic predictive factors noted in the general surgery and TKA models in this review suggest that non-clinical factors like day of the week, hospital type, time of surgery, clinician experience, and patient factors may be important considerations in predicting extended LOS^51^ as noted by others,^62,63^ albeit infrequently used as evident in the current review. In a recent scoping review, qualitative variables like patient incompliance, patient experience, and clinician rapport were also suggested as an important causal influence on prolonged LOS,^62^ which were not reported in this review.

Consensus on a consistent set of predictor variables in LOS models could assist the ability of researcher groups across the world to conduct external validations and work towards establishing transportable models predicting risk of prolonged LOS. Based on the frequency of the variables used in the studies included in the current review, we recommend that LOS prediction in surgical populations includes procedure-specific risk factors (type of surgery and technique) along with consideration of more universal factors like demographics (age > 75 and sex), comorbidity burden (ASA/CCI/APR-DRG scores), and admission characteristics (mode/day of admission).

In another systematic review looking at factors associated with extended LOS in TKA group,^64^ many common variables were noted with our findings. Consequently, we suggest that LOS prediction models in TKA should include demographic factors (age > 75, female sex, and BMI > 30), comorbidity risk score (ASA score >2 and frailty risk), procedure considerations (e.g. type of implant and technique), biological/physiological parameters (preoperative haemoglobin <130 g/L and serum albumin/creatinine), and admission characteristics (day of admission).

### Strengths and limitations

The validated PROBAST quality assessment of the included studies was a strength of our review. It revealed a significant gap in adoption of the TRIPOD reporting guidelines for prediction studies, leading to poor reporting, reflected as moderate to high ROB, impacting implementation and external validation feasibility. Many recent publications have implored the research community to attempt external validation before developing new models while accepting the evident challenges in reporting and reproducibility.^60,65^ This review further strengthens this imperative to improve the reporting in prognostic prediction modelling studies.

Majority of the data sources in our systematic review were classified as secondary data sources. As per the PROBAST tool recommendation, secondary data sources are considered as high ROB due to lack of data collection protocols, increasing the uncertainty about data validity^66^ and being event-based, limiting generalizability. Secondary data use is critical for long-term real-world evaluation of health interventions and system efficacy for optimal delivery of care and allows continuous improvement and monitoring of health care service delivery.^67^ Transparent reporting of data quality issues like missingness, inaccuracy, and inconsistency can assist in providing some reassurance that routinely collected data can be used as a strategic resource for research to improve health system efficiencies and effectiveness.^66,68,69^ We suggest that data hubs and repositories adopt evidence-based standardized frameworks to guide their data governance and evaluation practices^67,70^ to ensure transferability and generalization of results of secondary analysis of routinely collected health data. Pre-registration of studies and adherence to reporting guidelines for studies using such data is one of the strategies to reduce researcher bias.^71^

Finally, a comparison between the LOS medians and percentiles across the study populations could have improved the understanding of the heterogeneity across different hospital systems and countries. However, these data were inconsistently and incompletely reported, making such comparison difficult.

Due to the small number of validation studies, traditional meta-analysis methods may show convergence and yield unreliable estimates of prediction intervals.^72^ Also, we were unable to restore missing information from the primary studies to enable harmonizing or estimation of the model performance metrics.^73^ The use of Bayesian estimation framework has been recommended^73^ but lacks widespread adoption. Therefore, the results of the meta-analysis warrant further updating via further validation of this prediction model as well as testing of other models using IPD approaches.

## Conclusion

To the best of our knowledge, this is the first systematic review assessing quality of risk prediction models for hospital LOS in general surgery and TKA groups. We have shown that risk prediction models for prolonged LOS in general surgery and TKA populations were infrequently externally validated. Both statistical and ML methods appear to show acceptable to good discrimination performance, which is promising for future clinical utility and resource planning. However, study quality was generally poor, commonly related to poor reporting and lack of adoption of existing guidelines. Moving forward, a focus on unified reporting and analytic methods along with external validation is needed before clinical application.

## Supplemental Material

sj-docx-1-dhj-10.1177_20552076231177497 - Supplemental material for Hospital length of stay prediction for general surgery and total knee arthroplasty admissions: Systematic review and meta-analysis of published prediction models Click here for additional data file. Supplemental material, sj-docx-1-dhj-10.1177_20552076231177497 for Hospital length of stay prediction for general surgery and total knee arthroplasty admissions: Systematic review and meta-analysis of published prediction models by Swapna Gokhale, David Taylor, Jaskirath Gill, Yanan Hu, Nikolajs Zeps, Vincent Lequertier, Helena Teede and Joanne Enticott in DIGITAL HEALTH
