# Supplementary material for: Hospital length of stay prediction for general surgery and total knee arthroplasty admissions: Systematic review and meta-analysis of published prediction models
Source: Digit Health. 2023 May 29;9:20552076231177497. doi: 10.1177/20552076231177497 (PMC10240873; doi:10.1177/20552076231177497)
Supplement: sj-docx-1-dhj-10.1177_20552076231177497 - Supplemental material for Hospital length of stay prediction for general surgery and total knee arthroplasty admissions: Systematic review and meta-analysis of published prediction models [file sj-docx-1-dhj-10.1177_20552076231177497.docx]

**Supplementary file**

**Hospital Length of Stay Prediction Tools for General Surgery Populations and Total Knee Arthroplasty Admissions: Systematic Review and Meta-Analysis**

### Table S1 PRISMA checklist

| **Section and Topic** | **Item #** | **Checklist item** | **Location where item is reported (section)** |  |  |
| --- | --- | --- | --- | --- | --- |
| **TITLE** | | |  |  |  |
| Title | 1 | Identify the report as a systematic review. | Pg 1 |  |  |
| **ABSTRACT** | | |  |  |  |
| Abstract | 2 | See the PRISMA 2020 for Abstracts checklist. | Pg 2 |  |  |
| **INTRODUCTION** | | |  |  |  |
| Rationale | 3 | Describe the rationale for the review in the context of existing knowledge. | Pg 3,4 |  |  |
| Objectives | 4 | Provide an explicit statement of the objective(s) or question(s) the review addresses. | Pg 3,4 |  |  |
| **METHODS** | | |  |  |  |
| Eligibility criteria | 5 | Specify the inclusion and exclusion criteria for the review and how studies were grouped for the syntheses. | Pg 5 |  |  |
| Information sources | 6 | Specify all databases, registers, websites, organisations, reference lists and other sources searched or consulted to identify studies. Specify the date when each source was last searched or consulted. | Pg 5 |  |  |
| Search strategy | 7 | Present the full search strategies for all databases, registers and websites, including any filters and limits used. | Pg 6 |  |  |
| Selection process | 8 | Specify the methods used to decide whether a study met the inclusion criteria of the review, including how many reviewers screened each record and each report retrieved, whether they worked independently, and if applicable, details of automation tools used in the process. | Pg 6 |  |  |
| Data collection process | 9 | Specify the methods used to collect data from reports, including how many reviewers collected data from each report, whether they worked independently, any processes for obtaining or confirming data from study investigators, and if applicable, details of automation tools used in the process. | Pg 6 |  |  |
| Data items | 10a | List and define all outcomes for which data were sought. Specify whether all results that were compatible with each outcome domain in each study were sought (e.g. for all measures, time points, analyses), and if not, the methods used to decide which results to collect. | Supplement Table S3 |  |  |
|  | 10b | List and define all other variables for which data were sought (e.g. participant and intervention characteristics, funding sources). Describe any assumptions made about any missing or unclear information. | Supplement Table S3 |  |  |
| Study risk of bias assessment | 11 | Specify the methods used to assess risk of bias in the included studies, including details of the tool(s) used, how many reviewers assessed each study and whether they worked independently, and if applicable, details of automation tools used in the process. | Pg 6 |  |  |
| Effect measures | 12 | Specify for each outcome the effect measure(s) (e.g. Risk ratio, mean difference) used in the synthesis or presentation of results. | Pg 6 |  |  |
| Synthesis methods | 13a | Describe the processes used to decide which studies were eligible for each synthesis (e.g. tabulating the study intervention characteristics and comparing against the planned groups for each synthesis (item #5)). | Pg 6 |  |  |
|  | 13b | Describe any methods required to prepare the data for presentation or synthesis, such as handling of missing summary statistics, or data conversions. | Pg 6,7 |  |  |
|  | 13c | Describe any methods used to tabulate or visually display results of individual studies and syntheses. | Pg 6,7 |  |  |
|  | 13d | Describe any methods used to synthesize results and provide a rationale for the choice(s). If meta-analysis was performed, describe the model(s), method(s) to identify the presence and extent of statistical heterogeneity, and software package(s) used. | Pg 7,8 |  |  |
|  | 13e | Describe any methods used to explore possible causes of heterogeneity among study results (e.g. subgroup analysis, meta-regression). | Pg 7,8 |  |  |
|  | 13f | Describe any sensitivity analyses conducted to assess robustness of the synthesized results. | Pg 7,8 |  |  |
| Reporting bias assessment | 14 | Describe any methods used to assess risk of bias due to missing results in a synthesis (arising from reporting biases). | Pg 7,8 |  |  |
| Certainty assessment | 15 | Describe any methods used to assess certainty (or confidence) in the body of evidence for an outcome. | Pg 7,8 |  |  |
| **RESULTS** | | |  |  |  |
| Study selection | 16a | Describe the results of the search and selection process, from the number of records identified in the search to the number of studies included in the review, ideally using a flow diagram. | Pg 8,9 |  |  |
|  | 16b | Cite studies that might appear to meet the inclusion criteria, but which were excluded, and explain why they were excluded. | PRISMA flowchart Fig 1 |  |  |
| Study characteristics | 17 | Cite each included study and present its characteristics. | Table 2 and Supplement table S5 |  |  |
| Risk of bias in studies | 18 | Present assessments of risk of bias for each included study. | Table 4 and Supp table S7 |  |  |
| Results of individual studies | 19 | For all outcomes, present, for each study: (a) summary statistics for each group (where appropriate) and (b) an effect estimate and its precision (e.g. confidence/credible interval), ideally using structured tables or plots. | Supplement table S5 |  |  |
| Results of syntheses | 20a | For each synthesis, briefly summarise the characteristics and risk of bias among contributing studies. | Pg 9-18 |  |  |
|  | 20b | Present results of all statistical syntheses conducted. If meta-analysis was done, present for each the summary estimate and its precision (e.g. confidence/credible interval) and measures of statistical heterogeneity. If comparing groups, describe the direction of the effect. | Pg 9-18 |  |  |
|  | 20c | Present results of all investigations of possible causes of heterogeneity among study results. | Pg 9-18 |  |  |
|  | 20d | Present results of all sensitivity analyses conducted to assess the robustness of the synthesized results. | Pg 9-18 |  |  |
| Reporting biases | 21 | Present assessments of risk of bias due to missing results (arising from reporting biases) for each synthesis assessed. | Pg 9-18 |  |  |
| Certainty of evidence | 22 | Present assessments of certainty (or confidence) in the body of evidence for each outcome assessed. | Pg 9-18 |  |  |
| **DISCUSSION** | | |  |  |  |
| Discussion | 23a | Provide a general interpretation of the results in the context of other evidence. | Pg 18-22 |  |  |
|  | 23b | Discuss any limitations of the evidence included in the review. | Pg 18-22 |  |  |
|  | 23c | Discuss any limitations of the review processes used. | Pg 18-22 |  |  |
|  | 23d | Discuss implications of the results for practice, policy, and future research. | Pg 18-22 |  |  |
| **OTHER INFORMATION** | | |  |  |  |
| Registration and protocol | 24a | Provide registration information for the review, including register name and registration number, or state that the review was not registered. | Pg 3-5 |  |  |
|  | 24b | Indicate where the review protocol can be accessed, or state that a protocol was not prepared. | Pg 3-5 |  |  |
|  | 24c | Describe and explain any amendments to information provided at registration or in the protocol. | N/A |  |  |
| Support | 25 | Describe sources of financial or non-financial support for the review, and the role of the funders or sponsors in the review. | Pg 22-23 |  |  |
| Competing interests | 26 | Declare any competing interests of review authors. | Pg 22-23 |  |  |
| Availability of data, code and other materials | 27 | Report which of the following are publicly available and where they can be found template data collection forms; data extracted from included studies; data used for all analyses; analytic code; any other materials used in the review. | Supplementary Information |  |  |

###

### Table S2: Systematic Database Search strategy

| **Ovid MEDLINE(R) and Epub Ahead of Print, In-Process, In-Data-Review & Other Non-Indexed Citations, Daily and Versions 1946 to March 21, 2022** | **Embase Classic+ Embase 1947 to 2022 March 21** | **Cochrane** | **CINHAL** |
| --- | --- | --- | --- |
| 1 Risk Assessment/ 297904 2 (risk adj1 (assess* or analys* or adjust*)).ti,ab,mp. 360526 3 "Severity of Illness Index"/ 266438 4 "Predictive Value of Tests"/ 220311 5 Risk Adjustment/ 4061 6 risk factors/ 915390 7 risk predict* factor*.ti,ab,mp. 30 8 Electronic Health Records/ 24661 9 Electronic Health Record*.ti,ab,mp. 33114 10 electronic medical record*.ti,ab,mp. 17689 11 electronic patient record*.ti,ab,mp. 2132 12 1 or 2 or 3 or 4 or 5 or 6 or 7 or 8 or 9 or 10 or 11 1573324 13 Regression Analysis/ 133531 14 exp Artificial Intelligence/ 139940 15 machine learning.ti,ab,mp. 43838 16 "Models, Statistical"/ 97702 17 Data Mining/ 10062 18 Logistic Models/ 149901 19 Computer Simulation/ 203426 20 computer simulation.ti,ab,mp. 207350 21 predict*.ti. 326499 22 (risk predict* adj1 (tool* or model*)).ti,ab,mp. 3612 23 (risk adjust* adj1 (tool* or model*)).ti,ab,mp. 758 24 13 or 14 or 15 or 16 or 17 or 18 or 19 or 20 or 21 or 22 or 23 967880 25 exp "Length of Stay"/ 98712 26 ((unplanned or unexpected or extended) adj4 stay*).ti,ab. 1411 27 (care adj1 days).ti,ab. 236 28 (length* adj2 stay*).ti,mp. 129263 29 25 or 26 or 27 or 28 129958 | 1. risk assessment/  2. (risk adj1 (assess* or analys* or adjust*)).ti,ab,mp.  3. "Severity of Illness Index"/  4. "Predictive value of tests"/  5. "prediction and forecasting"/  6. risk factors/  7. risk predict* factor*.ti,ab,mp.  8. Electronic Health Records/  9. medical record/ or electronic patient record/ or electronic medical record/  10. electronic medical record*.ti,ab,mp.  11. electronic patient record*.ti,ab,mp.  12. 1 or 2 or 3 or 4 or 5 or 6 or 7 or 8 or 9 or 10 or 11  13. regression analysis/  14. artificial intelligence/  15. machine learning/  16. statistical model/  17. Data Mining/  18. computer simulation/  19. computer simulation.ti,ab.mp  20. predict*.ti.  21. (risk predict* adj1 (tool* or model*)).ti,ab,mp.  22. (risk adjust* adj1 (tool* or model*)).ti,ab,mp.  23. 13 or 14 or 15 or 16 or 17 or 18 or 19 or 20 or 21 or 22  24. exp "length of stay"/  25. ((unplanned or unexpected or extended) adj4 stay*).ti,ab.  26. (care adj1 days).ti,ab.  27. (length* adj2 stay*).ti,mp.  28. 24 or 25 or 26 or 27  29. 12 and 23 and 28 | MeSH descriptor: [Risk Assessment] this term only  (risk Near/1 (assess* or analys* or adjust*)):ti,ab,kw  MeSH descriptor: [Severity of Illness Index] this term only  MeSH descriptor: [Predictive Value of Tests] this term only  MeSH descriptor: [Risk Adjustment] this term only  MeSH descriptor: [Risk Factors] this term only  (risk predict* factor*):ti,ab,kw  MeSH descriptor: [Electronic Health Records] this term only  (electronic (health or medical or patient) record*):ti,ab,kw  #1 or #2 or #3 or #4 or #5 or #6 or #7 or #8 or #9  MeSH descriptor: [Regression Analysis] this term only  MeSH descriptor: [Artificial Intelligence] explode all trees  (machine learning):ti,ab,kw  MeSH descriptor: [Models, Statistical] this term only  MeSH descriptor: [Data Mining] this term only  MeSH descriptor: [Logistic Models] this term only  MeSH descriptor: [Computer Simulation] this term only  (Computer Simulation):ti,ab,kw  (predict*):ti  ((risk predict* Near/1 (tool* or model*))):ti,ab,kw  ((risk adjust* Near/1 (tool* or model*))):ti,ab,kw  #11 or #12 or #13 or #14 or #15 or #16 or #17 or #18 or #19 or #20 or #21  MeSH descriptor: [Length of Stay] explode all trees  ((unplanned or unexpected or extended) NEAR/4 stay):ti,ab,kw  (care Near/1 days):ti,ab,kw  ((length near/2 stay)):ti OR ((length near/2 stay)):kw  #23 or #24 or #25 or #26  #10 and #22 and #27 | (MH "Risk Assessment")  TI ( (risk N1 (assess* or analys* or adjust*)) or AB ( (risk N1 (assess* or analys* or adjust*))  (MH "Severity of Illness Indices")  (MH "Predictive Value of Tests")  TI "Risk Adjustment" or AB "Risk Adjustment"  (MH "Risk Factors")  TI "risk predict* factor*" or AB "risk predict* factor*"  (MH "Electronic Health Records")  TI ( electronic health record* or electronic medical record* or emr or ehr)  AB ( electronic health record* or electronic medical record* or emr or ehr )  S1 OR S2 OR S3 OR S4 OR S5 OR S6 OR S7 OR S8 OR S9 OR S10  (MH "Regression")  (MH "Artificial Intelligence")  TI machine learning OR AB machine learning  (MH "Models, Statistical")  (MH "Data Mining")  TI logistic model* OR AB logistic model*  (MH "Computer Simulation")  TI computer simulation OR AB computer simulation  TI predict*  TI ( (risk predict* n1 (tool* or model*)) ) OR AB ( (risk predict* n1 (tool* or model*)) )  TI ( (risk adjust* n1 (tool* or model*)) ) OR AB ( (risk predict* n1 (tool* or model*)) )  S12 OR S13 OR S14 OR S15 OR S16 OR S17 OR S18 OR S19 OR S20 OR S21 OR S22  (MH "Length of Stay")  TI (((unplanned or unexpected or extended) n4 stay*) ) OR AB ( ((unplanned or unexpected or extended) n4 stay*) )  TI care n1 days OR AB care n1 days  TI (length* n2 stay*)  S24 OR S25 OR S26 OR S27  S11 AND S23 AND S28 |

### Table S3: Inclusion/exclusion criteria

|  | **Inclusion** | **Exclusion** |
| --- | --- | --- |
| Population | Adults admitted to acute hospital care  Studies from OECD countries  General Surgery admissions  Total knee arthroplasty admissions | Newborn/infants/age<15 yrs old  Maternity admissions  Paediatric admissions  Other single diagnostic groups  Non-surgical admissions  Nursing home/community (rehabilitation) hospital admissions  All outpatient admissions |
| Intervention | Risk prediction tools/models intended to aid decision support preadmission, on admission or post discharge.  Evidence of predictive utility reported in the form of prediction performance metrics like discriminations, calibration, and overall model accuracy | Studies reporting association metrics only between dependant variable (LOS) and variables/features tested |
| Outcomes | Length of stay (LOS) prediction in days/hours (either unit of measurement was accepted as they could be combined for calculation if required) | LOS<24 hours/day admissions  Cumulative/aggregate measures of LOS  Studies reporting other outcomes like mortality/complications etc as a proxy for prolonged LOS |

### Table S4: List of data items extracted for each included study:

| - Study Author |
| --- |
| - Title |
| - Year of publication |
| - Lead authors contact details |
| - Country in which the study conducted |
| - Study funding sources |
| - Possible conflicts of interest for study authors |
| - Aim of study |
| - Study design |
| - Study Temporality |
| - Population description (from which study participants are drawn) |
| - Setting (include location and social context) |
| - Study Inclusion criteria |
| - Study Exclusion criteria |
| - Diagnostic groups |
| - Data Source |
| - Total number of participants |
| - Total number of admissions |
| - Study Duration (Years) |
| - Number of Candidate predictors |
| - Type of predictors used: 1) administrative, 2) demographic and anthropometric, 3) diagnosis (primary and secondary including comorbidities) and procedure type, 4) physical examination (biological and physiological parameters), 5) risk scores (e.g., Frailty Index, Charlson Comorbidity Index (CCI)), 6) admission characteristics, 7) hospital characteristics, 8) healthcare professional characteristics, 9) documentation and clinical notes and 10) medications. |
| - Timing of predictor measurement: Admission/Preadmission, During hospitalisation, Discharge/Post discharge |
| - Data analysis/modelling method used: classical statistical methods, machine learning (ML) or both |
| - Details of ML method used |
| - Method for selection of predictors for inclusion in multivariable modelling |
| - Method for selection of predictors during multivariable modelling like full model approach, backward or forward selection and criteria used (e.g., p-value, Akaike Information Criterion) |
| - Shrinkage of predictor weights or regression coefficients (e.g., no shrinkage, uniform shrinkage, penalized estimation) |
| - Handling of missing data: excluded/imputed/complete case analysis/other |
| - Transformation done of LOS. E.g., log/square root |
| - Hyperparameter tuning and selection methods |
| - Calibration (calibration plot, calibration slope, Hosmer-Lemeshaw test) |
| - Classification / Discrimination measures (e.g., Area under Receiver Operating Curve (AUROC), C index sensitivity, specificity, predictive values, net reclassification improvement) and whether a priori cut points were used |
| - Overall Measures/goodness of fit measures (Mean Square Error (MSE), Root Mean Square Error (RMSE), Brier score, R2 statistic) |
| - Method used for testing and optimising model performance: development dataset only (random split of data, resampling methods, e.g., bootstrap or cross-validation, none) or separate external validation (e.g., temporal, geographical, different setting, diff |
| - In case of poor validation, whether model was adjusted or updated (e.g., intercept recalibrated, predictor effects adjusted, or new predictors added) |
| - Was the model externally validated? |
| - Was there any report of implementation and change in clinical practice |
| - LOS measured as Continuous/Categorical |
| - Other outcomes |
| - Prolonged LOS cut off levels for definition of LOS |
| - Key conclusions of study authors: interpretation of presented models- useful for practice vs exploratory, comparison with other studies, strengths, and limitations |
| - References to other relevant studies |
| - Correspondence required for further study information |

### Table S5a: Study characteristics of 15 included studies (Country, Study Temporality, Study design, Diagnostic Group, setting (include location and social context), Study Duration (Years))

| **Study Author, Year** | **Country in which the study conducted** | **Study Temporality** | **Study design** | **Diagnostic Group** | **Setting (include location and social context)** | **Study Duration (Years)** |
| --- | --- | --- | --- | --- | --- | --- |
| Almeida 2013 | Other: Portugal | prospective | Cohort study | General Surgery | University Hospital large General Surgery Department in Lisbon, Portugal. | 0.7 |
| Anis 2020 | United States | prospective | Cohort study | TKA | patients at academic institutions and different volume hospitals from two different states (Ohio and Florida) | 4 |
| Carter 2014 | United Kingdom (UK) | retrospective | Cohort study | TKA | National Health Service (NHS) tertiary referral centre for specialist orthopaedic services | 4 |
| Cunic 2014 | Canada | retrospective | Cohort study | TKA | Kingston General Hospital Ontario, Canada | 2.8 |
| Jorgensen 2016 | Other: Denmark | prospective | Cohort study | TKA | registry collecting data from 9 different dedicated arthroplasty units across Denmark | 2.5 |
| Jorgensen 2021 | Other: Denmark | prospective | Cohort study | TKA | registry collecting data from 9 different dedicated arthroplasty units across Denmark | 1.7 |
| McIsaac 2020 | Canada | prospective | Cohort study | General Surgery | academic health sciences network (the Ottawa Hospital) and a community hospital (Montfort) | 2.9 |
| Navarro 2018 | United States | retrospective | Cohort study | TKA | Non- federal acute care hospitals in New York | 7 |
| Piuzzi 2019 | United States | prospective | Cohort study | TKA | 4 facilities in a single health-care system, all Medicare funded patients in Cleveland clinic Ortho group OHIO USA | 1.6 |
| Ramkumar 2019 | United States | retrospective | Cohort study | TKA | 11 main and satellite hospitals at the senior author's institution in Cleveland, Ohio | 5 |
| Sephton 2020 | UK | retrospective | Cohort study | TKA | Imperial College Healthcare NHS Trust, Charing Cross Hospital, London, | 3 |
| Sessler 2010 | United States | retrospective | Cohort study | General Surgery | MEDPAR (Medicare or Centres for Medicare and Medicaid Services) claims database in US | 5 |
| Sigakis 2013 | United States | retrospective | Cohort study | General Surgery | 34 hospitals from nine countries across Europe, Australia, the UK and USA | 5 |
| Sutherland 2019 | Canada | prospective | Cohort study | General Surgery | Vancouver, British Columbia (BC), Canada. | 4 |
| Winemaker 2015 | Canada | Cross sectional study | Cross sectional study | TKA | high-volume academic arthroplasty centre in Ontario, Canada | 2 |

### Table S5b: Study characteristics of 15 included studies (Sample size, Study Inclusion criteria, Study Exclusion criteria, Primary Outcome LOS definition, Timing of predictor measurement, Type of predictors used)

| **Study Author, Year** | **Final Sample size** | **Study Inclusion criteria** | **Study Exclusion criteria** | **Primary Outcome LOS definition** | **Timing of predictor measurement/prediction** | **Type of predictors used (Predictor Categories)** |
| --- | --- | --- | --- | --- | --- | --- |
| Almeida 2013 | 300 | adult patients (18 years)  predicted length of stay >=4 d | organ transplantation, coma, bedridden, intensive care patients, or previous surgery, chemo/radiotherapy during the year before hospital admission; patients submitted to surgery before nutritional assessment were also excluded | LOS >10 | Admission; Other: Discharge | Demographics and anthropometric; Risk score EG CCI/CFI etc; Other: Nutritional risk scores: NRS2002, MUST, SGA, BMI loss |
| Anis 2020 | 5,663 | TKA/Unilateral Knee Arthroplasty at large integrated health system | emergent or nonelective arthroplasty, tumour as an indication for surgery, language barrier, and ipsilateral revision arthroplasty | LOS (continuous) | Preadmission | Demographics and anthropometric; Diagnoses and Medical history (includes Co-morbidities); Physical examination: Biological and Physiological Parameters; Risk score EG CCI/CFS etc |
| Carter 2014 | 2,130 | primary TKA, Adult >15 yrs old | secondary TKA | LOS (continuous) | Admission; Other: data was examined retrospectively hence other predictor timing is not clear | Administrative data; Demographics and anthropometric; Diagnoses and Medical history (includes Co-morbidities); Timing and frequency of admission |
| Cunic 2014 | 241 | primary elective arthroplasty | revision or non-elective arthroplasty | LOS > 5 d | Preadmission | Demographics and anthropometric; Diagnoses and Medical history (includes Co-morbidities); Risk score EG CCI/CFI etc |
| Jorgensen 2016 | 8,288 | consecutive unselected unilateral THA and TKA with LOS >4 days or 90 days readmission with potentially preventable complications (medical/surgical) refer to table 1 in text | patients with non-preventable post operative complications like post operative ileus, diarrhoea, post anaesthetic complications, non-traumatic periprosthetic fracture etc. refer to table 1 in text | LOS > 4 d | Preadmission | Demographics and anthropometric; Physical examination: Biological and Physiological Parameters |
| Jorgensen 2021 | 10,576 | consecutive unselected unilateral THA and TKA | age < 18 years,  simultaneous bilateral procedures,  procedures due to cancer or severe con-genital disorder, patients with major surgery on the lower extremities before or after 90 days from the elective procedure | LOS > 2 d | Preadmission; Admission | Demographics and anthropometric; Diagnoses and Medical history (includes Co-morbidities); Other: weekday of surgery |
| McIsaac 2020 | 645 | community dwelling adults>65yrs, having elective noncardiac surgery | the planned surgery was a revision procedure, the individual was unable to communicate in English or French, was unable to be reached by telephone, or was unable to respond to study questionnaires at baseline | LOS >5 d | Preadmission | Diagnoses and Medical history (include Co-morbidities); Risk score EG CCI/CFI etc; Other: 3 risk scores: Frailty index, Fried Phenotype and Clinical frailty score |
| Navarro 2018 | 1,41,446 | APR_DRG Code 302 Knee joint replacement and clinical classification software Procedure code 152 Arthroplasty Knee, | revision or partial knee arthroplasty, non-medicare patients | LOS 1-3 LOS 4-5  LOS >5 days | Other: post discharge | Administrative data; Diagnoses and Medical history (includes Co-morbidities); Risk score EG CCI/CFS etc |
| Piuzzi 2019 | 3,270 | primary TKA | revision of simultaneous bilat TKA, surgeon not enrolled in electronic data collection system, or pt. did not complete data collection form | LOS <=1 d | Admission | Administrative data; Demographics and anthropometric; Diagnoses and Medical history (includes Co-morbidities); Physical examination: Biological and Physiological Parameters; Timing and frequency of admission; Hospital Characteristics; Health care professional characteristics |
| Ramkumar 2019 | 1,71,025 | > 65 yrs age, Medicare-funded, inpatient TKA services and discharged between Jan 1, 2009 to Dec 31, 2013 | pts with missing data from NIS, patients with a LOS or inpatient charge greater than the 99th percentile or less than the 1st percentile were removed to adjust for outliers (2.5% were excluded) | LOS >=4 d | Admission | Administrative data; Demographics and anthropometric; Diagnoses and Medical history (includes Co-morbidities); Risk score EG CCI/CFI etc; Timing and frequency of admission; Hospital Characteristics; Other: APR Risk of mortality and APR severity of illness |
| Sephton 2020 | 155 | primary Unicompartmental Knee arthroplasty (UKA) | patients under-going bilateral UKAs or revision surgery | LOS >3 days | Admission; Other: perioperative factors | Demographics and anthropometric; Diagnoses and Medical history (includes Co-morbidities); Physical examination: Biological and Physiological Parameters; Risk score EG CCI/CFI etc; Health care professional characteristics |
| Sessler 2010 | 1,75,89,683 | age > 65, have had major admission to hospital costing>5000 USD, and follow up >1 yr | younger than 65 yr, those having no procedure or procedures with an annual average occurrence of less than 5,000, or a patient stay with less than 1 yr of follow-up. | LOS > 5 days | Admission | Demographics and anthropometric; Diagnoses and Medical history (includes Co-morbidities) |
| Sigakis 2013 | 1,08,423 | RSI: >18 yrs, inpatient admission that included anaesthetics between 2006-2011 at Massachusetts General Hospital |  | median LOS for the primary ICD-9-CM procedure code | Admission; Other: post-discharge | Diagnoses and Medical history (include Co-morbidities); Other: ICD9-CM procedural and diagnostic codes |
| Sutherland 2019 | 1,165 | Elective general surgery for benign conditions and whose scheduled surgery was at least two weeks from being assigned to the wait list | day surgery, not consenting to participation | LOS (continuous) | Preadmission | Demographics and anthropometric; Diagnoses and Medical history (includes Co-morbidities); Other: Patient reported outcomes |
| Winemaker 2015 | 1,459 | elective primary total hip or knee surgery and attended the preoperative clinic for medical history and assessment | incomplete or non-retrievable preoperative anaesthetic record or who underwent nonelective or revision TJR | LOS<=3 (reference category)  LOS = 4 LOS>=5 | Preadmission; Admission | Demographics and anthropometric; Diagnoses and Medical history (includes Co-morbidities); Physical examination: Biological and Physiological Parameters |

### Table S5c: Study characteristics of 15 included studies (Predictive modelling methods and Analytical Pipeline)

| **Study Author, Year** | **Data analysis/modelling method used** | **No.**  **of variables** | **Imputation of Missing Data** | **Method for selection of predictors for inclusion in multivariable modelling** | **Method for selection of predictors during multivariable modelling** | **Transformation done of LOS?** | **Hyperparameter tuning and selection?** | **Method used for testing and optimising model performance** |
| --- | --- | --- | --- | --- | --- | --- | --- | --- |
| Almeida 2013 | Classical statistical approach | 7 | not specified | all candidate predictors | not specified | no | n/a | not specified |
| Anis 2020 | Both | 16 | Yes, Multiple imputation by chained equations was implemented to handle missing data | all candidate predictors | Full models were reduced by sequentially removing the variable that causes the largest reduction in Akaike Information Criterion (AIC) until no reduction occurred to build the most parsimonious predictive model | not specified | not specified | Cross-validation of by randomly partitioning each Cohort into ten subsets after which the full imputation process was implemented within each subset followed by the modelling processes within each imputed dataset |
| Carter 2014 | Classical statistical approach | 7 | not specified | pre-selection based on unadjusted association with the outcome like univariable modelling | p value, forward selection | not specified | n/a | not specified |
| Cunic 2014 | Classical statistical approach | 2 | not specified | all candidate predictors | p value | not specified | n/a | not specified |
| Jorgensen 2016 | Classical statistical approach | 12 | not specified | pre-selection based on unadjusted association with the outcome like univariable modelling | p-value <=0.10 | no | n/a | not specified |
| Jorgensen 2021 | Classical statistical approach | 11 | No, participants with missing data were excluded (n= 589) | all candidate predictors | Directed Acyclic Graphs method (Shirer and Platt 2008) | not specified | n/a | not specified |
| McIsaac 2020 | Classical statistical approach | 7 | No, complete case analysis. patients with missing data excluded | all candidate predictors | full model approach, P value | not specified | n/a | 1000 bootstrap samples were created by drawing individuals with replacement from the full data set, sensitivity analysis |
| Navarro 2018 | Machine Learning Approach | 8 | not specified | all candidate predictors | full model approach | not specified | not specified | random split, train test and cross-validation |
| Piuzzi 2019 | Classical statistical approach | 8 | Yes, using multiple imputation by chained equations | all candidate predictors | full model approach followed by AIC | no | n/a | bootstrapping |
| Ramkumar 2019 | Machine Learning Approach | 20 | No, patients with missing data were excluded | all candidate predictors | not specified | no | not specified | random split, cross validation |
| Sephton 2020 | Classical statistical approach | 8 | not specified | all candidate predictors | p value | no | n/a | not specified |
| Sessler 2010 | Classical statistical approach | 1094 | No, patients with missing data were excluded | all candidate predictors | covariates were selected in a stepwise manner based on the statistical significance of the covariates in a multivariable model (Stepwise Hierarchical Selection) | no | n/a | random split of data, bootstrapping, external validation |
| Sigakis 2013 | Classical statistical approach | 1096 | not specified | all candidate predictors | not specified | not specified | n/a | sensitivity analysis by testing self-fulfilling ICD codes |
| Sutherland 2019 | Classical statistical approach | 12 | not specified | all candidate predictors | not specified | not specified | n/a | not specified |
| Winemaker 2015 | Classical statistical approach | 10 | not specified | all candidate predictors | Stepwise selection procedure with p <=0.2 | no | n/a | not specified |

### Table S6: Performance measures used in 15 included Studies

| **Study Author, Year** | **Diagnosis** | **Calibration** | **Classification / Discrimination measures** | **Overall Measures/goodness of fit measures** |
| --- | --- | --- | --- | --- |
| Almeida 2013 | General Surgery |  | sensitivity, specificity, PPV, NPV |  |
| McIsaac 2020 | General Surgery | Likelihood ratio x2 | AUROC, Continuous NRI | change in Nagelkerke's R2 |
| Sessler 2010 | General Surgery |  | C-statistic/C-index |  |
| Sigakis 2013 | General Surgery | Calibration plots | AUROC | Brier scores |
| Sutherland 2019 | General Surgery |  | AIC | Adjusted R-squared |
| Anis 2020 | TKA |  |  | RMSE and MAE |
| Carter 2014 | TKA |  | AIC | Accuracy |
| Cunic 2014 | TKA |  | AUROC, Specificity and sensitivity, PPV and NPV |  |
| Jorgensen 2016 | TKA |  | AUROC, PPV, NPV |  |
| Jorgensen 2021 | TKA |  | AUROC, Sensitivity and specificity | Accuracy |
| Navarro 2018 | TKA |  | AUROC | Accuracy |
| Piuzzi 2019 | TKA |  | C-statistic/C-index |  |
| Ramkumar 2019 | TKA |  | AUROC | Accuracy |
| Sephton 2020 | TKA | HL goodness of fit test |  | Nagelkerke’s R2 |
| Winemaker 2015 | TKA |  | AUROC |  |

Table S7: Risk of Bias Assessments for included TKA studies (n=10)

|  | **TYPE OF PREDICTION MODEL** | **PARTICIPANT RISK OF BIAS** | **PARTICIPANT APPLICABILITY** | **PREDICTOR RISK OF BIAS** | **PREDICTOR APPLICABILITY** | **OUTCOME RISK OF BIAS** | **OUTCOME APPLICABILITY** | **ANALYSIS RISK OF BIAS** | **OVERALL RISK OF BIAS** | **OVERALL APPLICABILITY CONCERN** |
| --- | --- | --- | --- | --- | --- | --- | --- | --- | --- | --- |
| **Anis 2020** | Development and Validation | 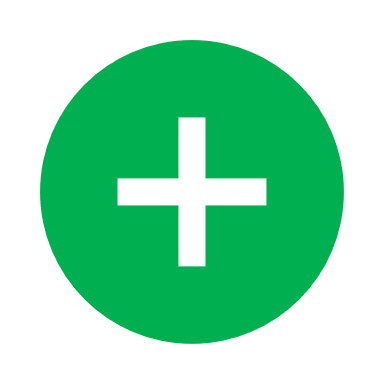 | 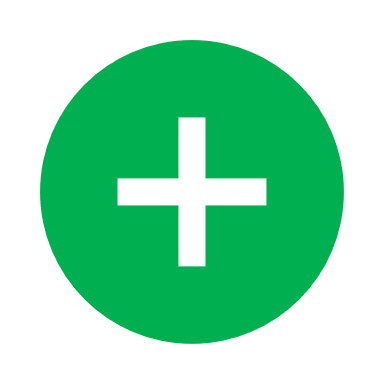 | 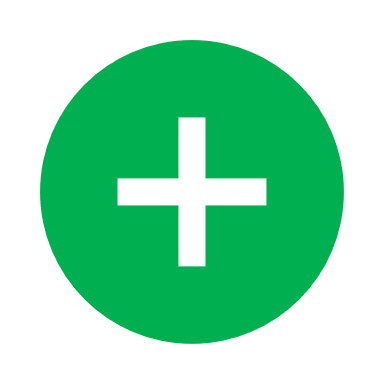 | 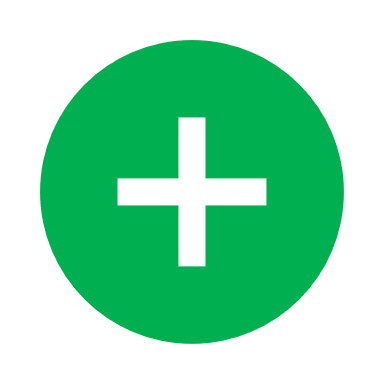 | 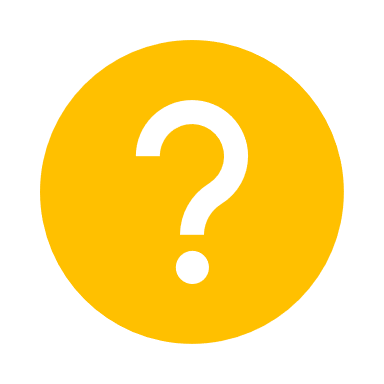 | 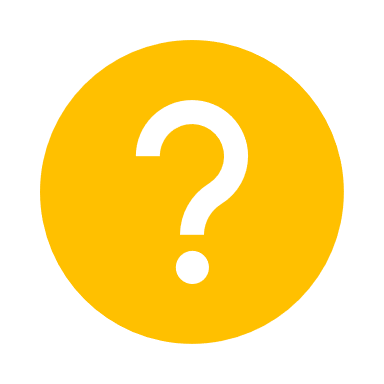 | 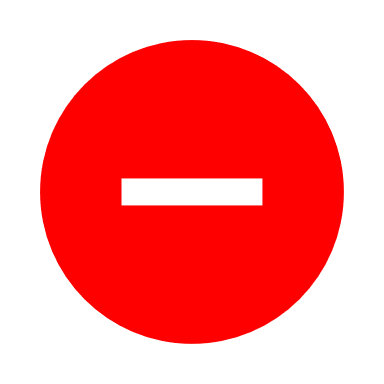 | 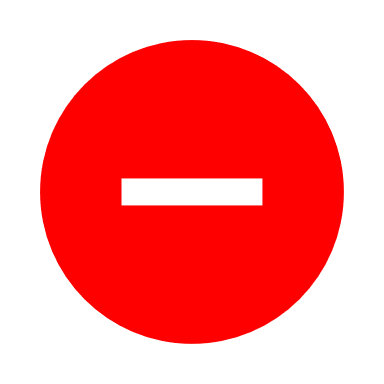 | **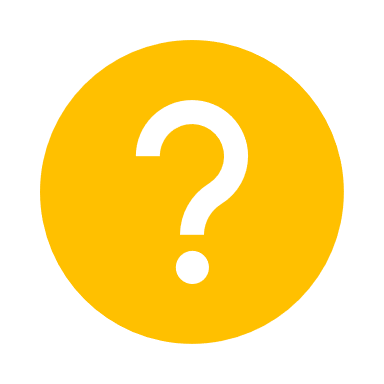** |
| **Carter 2014** | Development only | 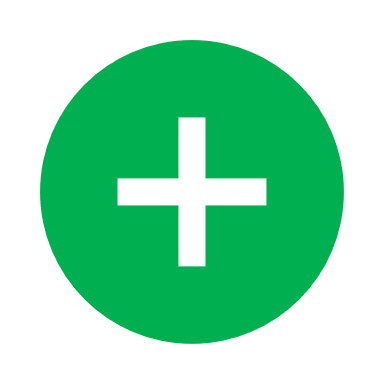 | 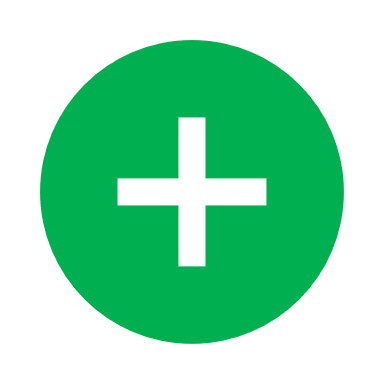 | 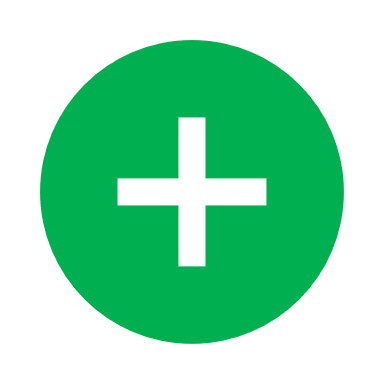 | 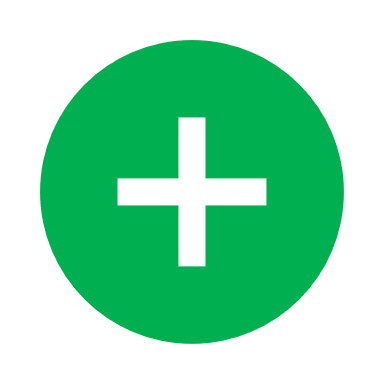 | 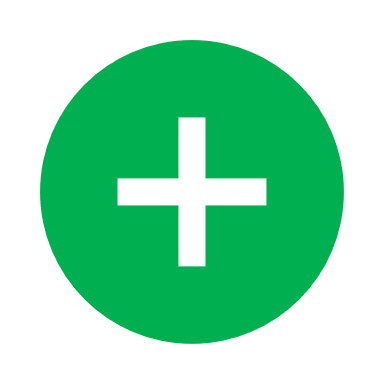 | 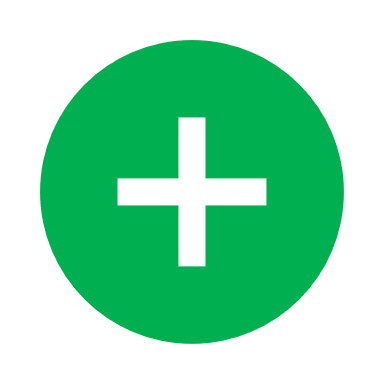 | 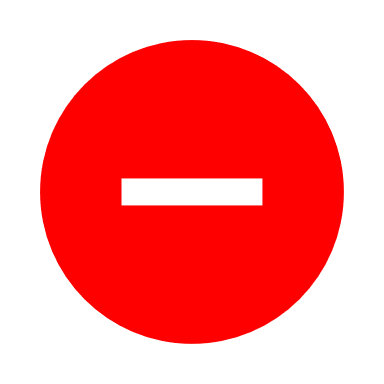 | 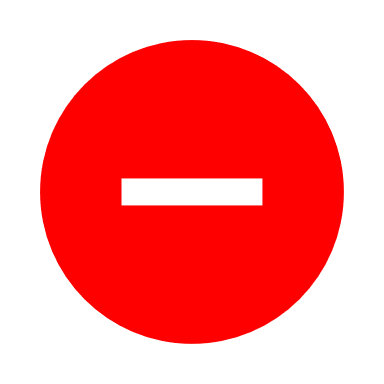 | **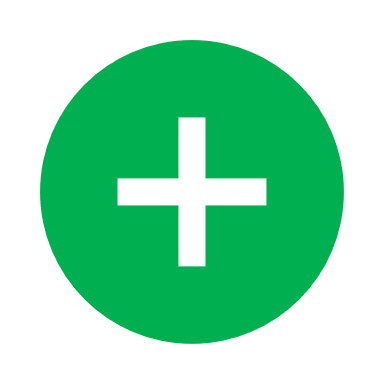** |
| **Cunic 2014** | Development only | 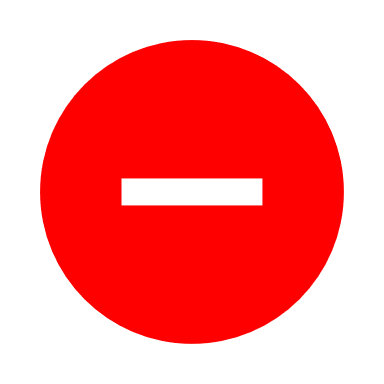 | 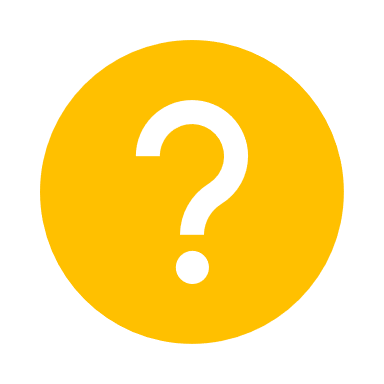 | 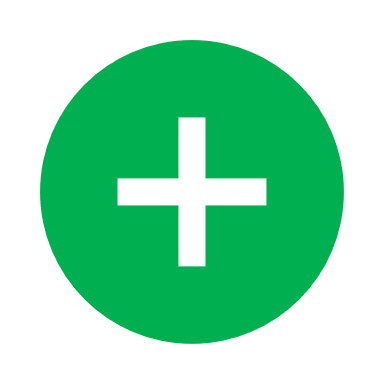 | 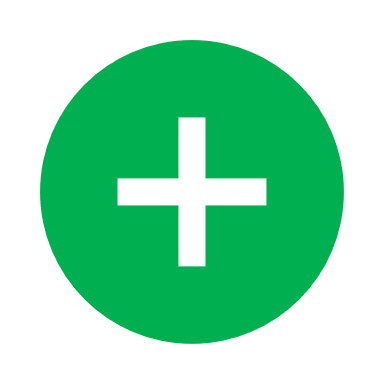 | 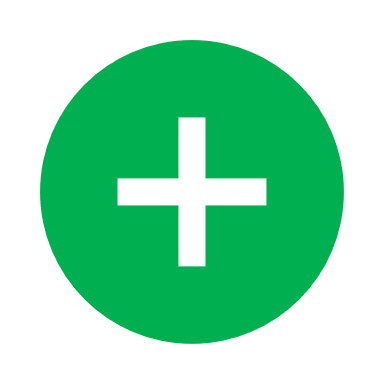 | 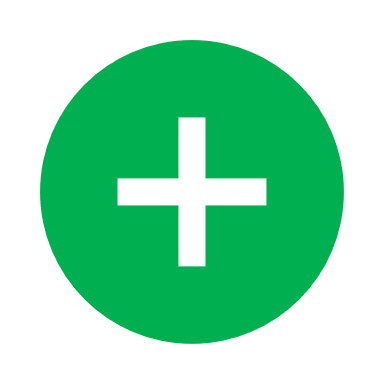 | 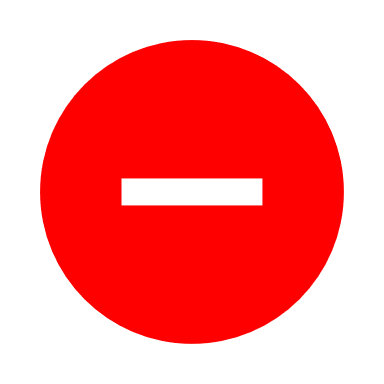 | 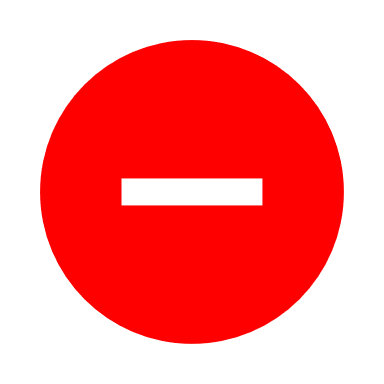 | **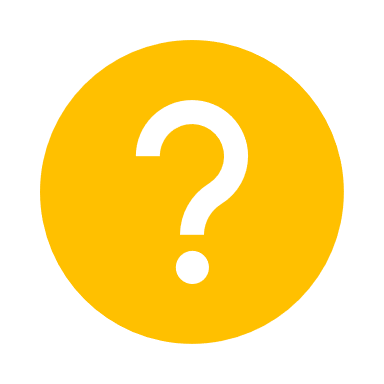** |
| **Jorgensen 2016** | Development only | 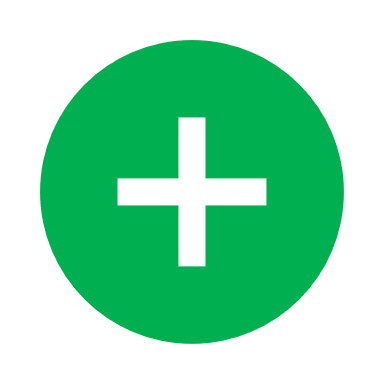 | 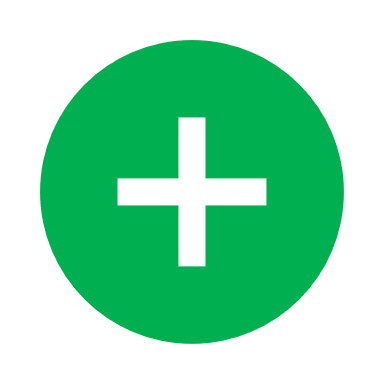 | 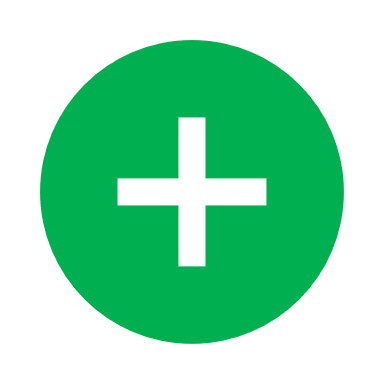 | 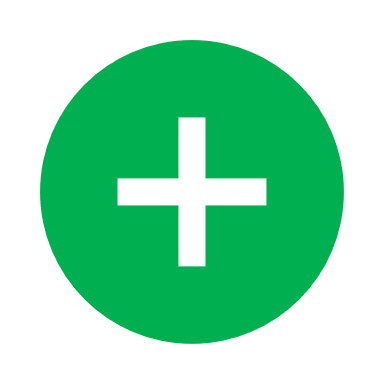 | 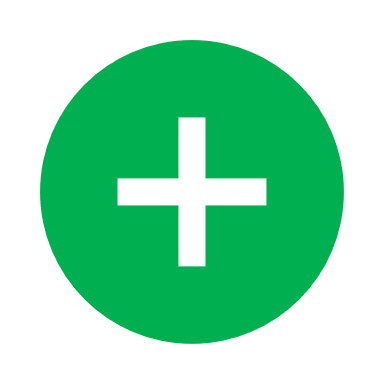 | 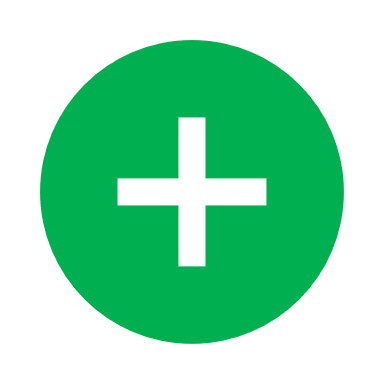 | 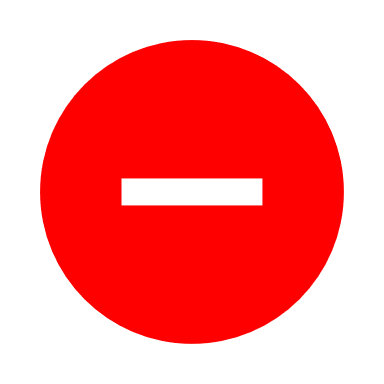 | 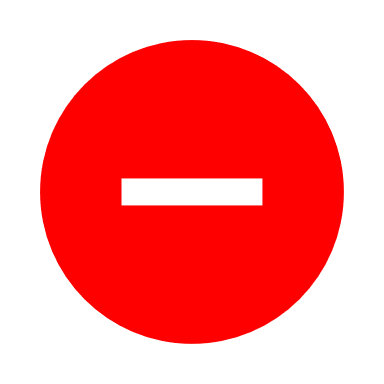 | **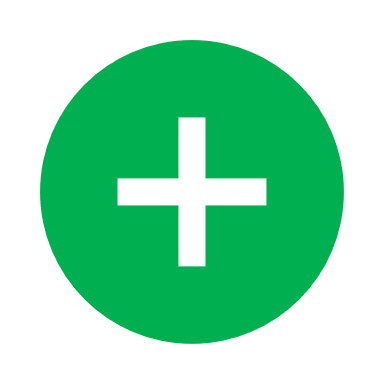** |
| **Jorgensen 2021** | Development only | 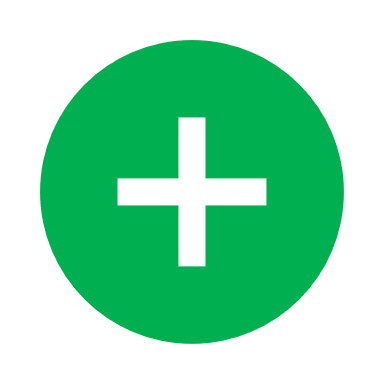 | 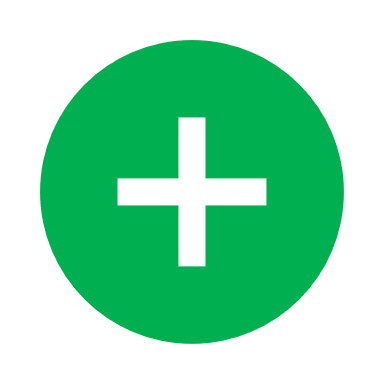 | 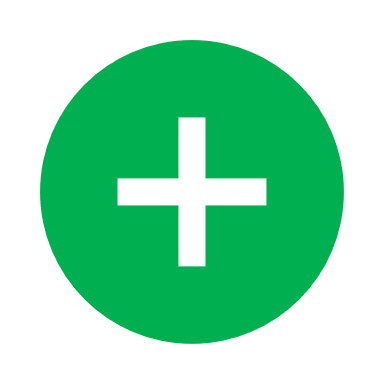 | 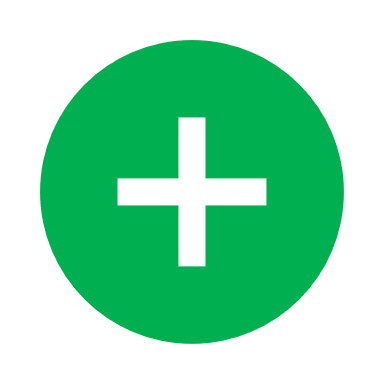 | 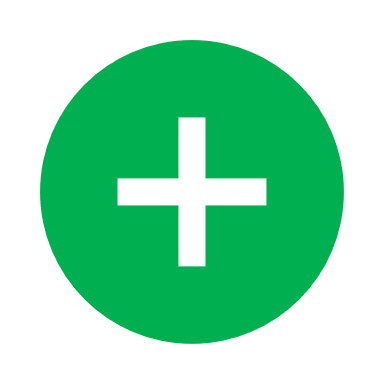 | 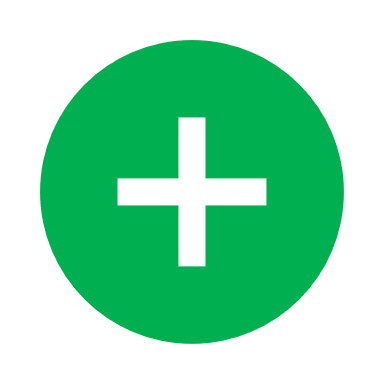 | 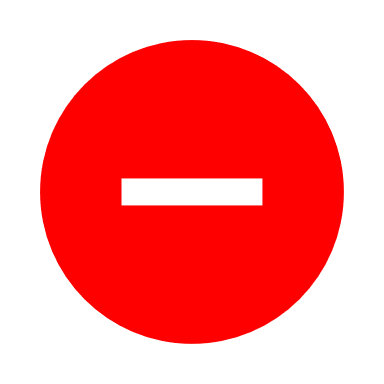 | 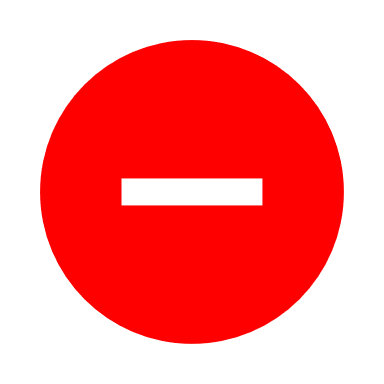 | **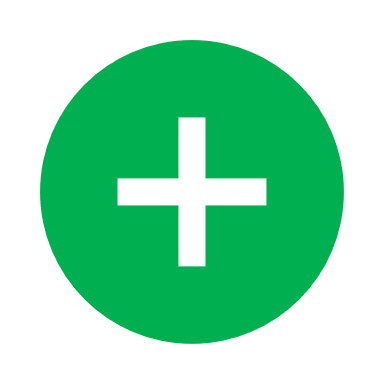** |
| **Navarro 2018** | Development only | 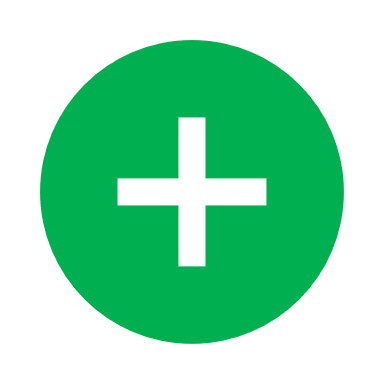 | 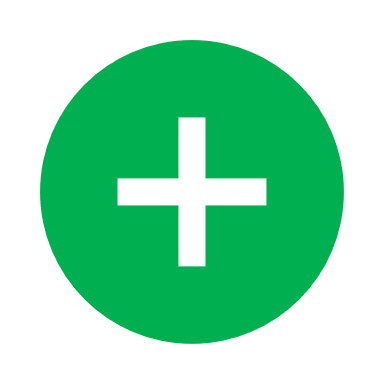 | 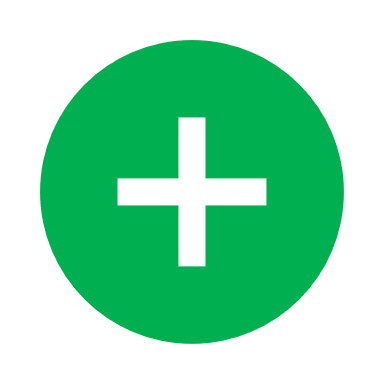 | 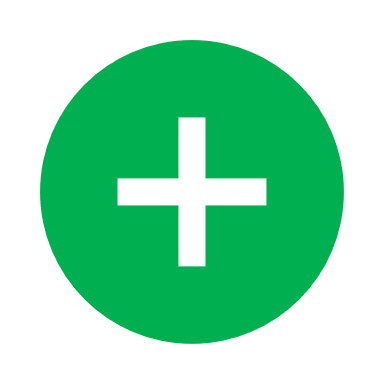 | 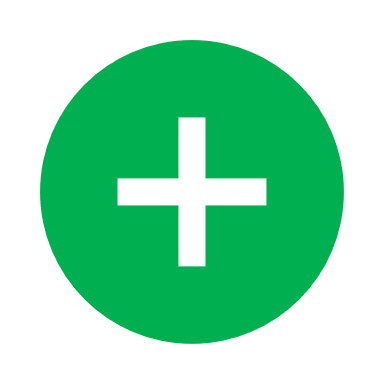 | 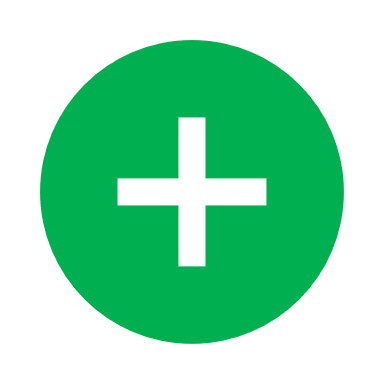 | 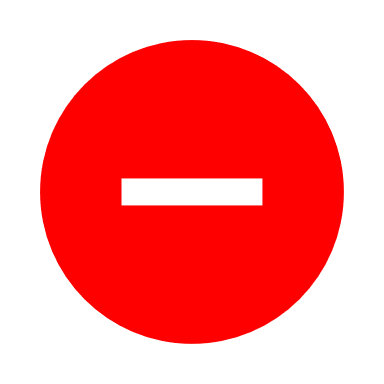 | 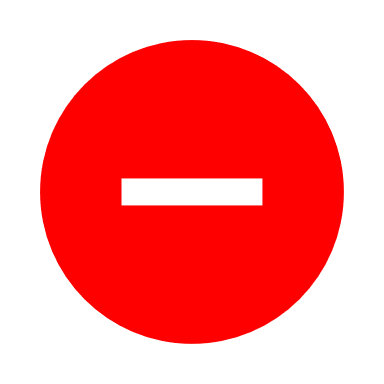 | **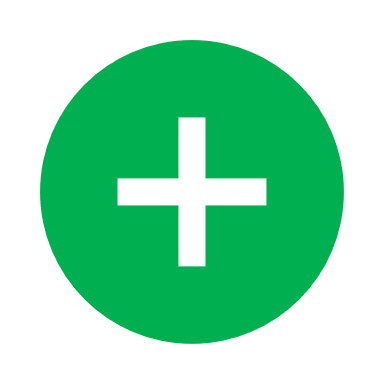** |
| **Piuzzi 2019** | Development only | 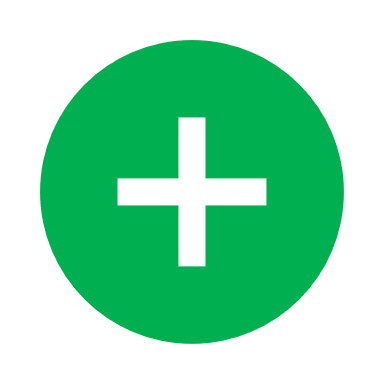 | 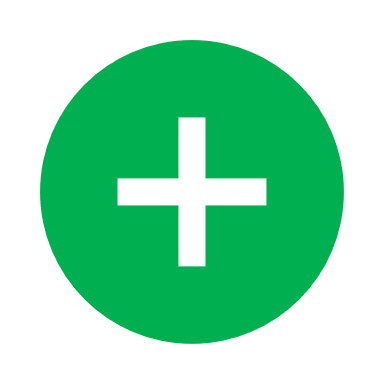 | 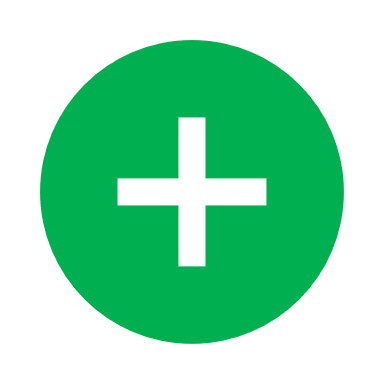 | 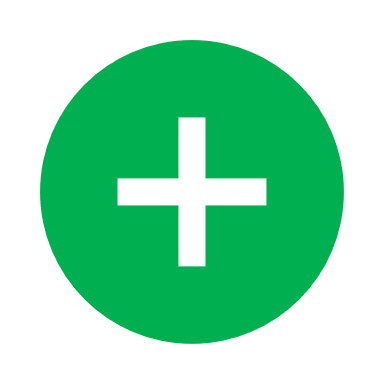 | 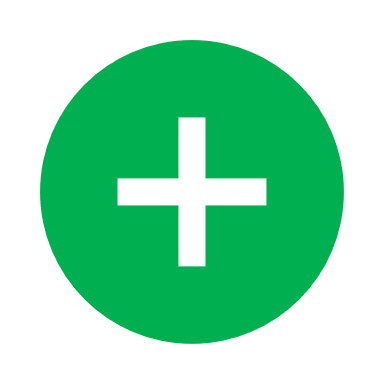 | 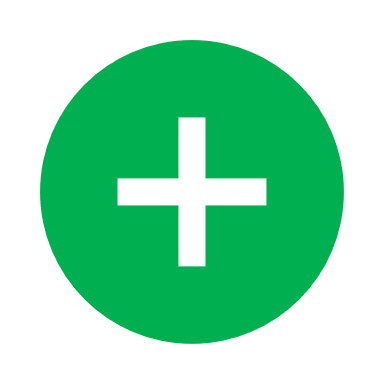 | 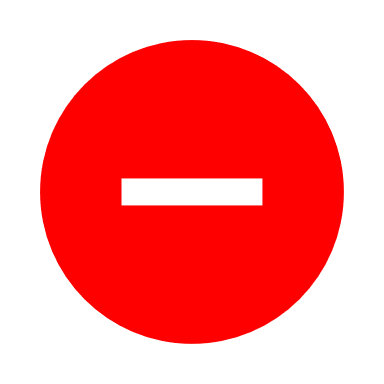 | 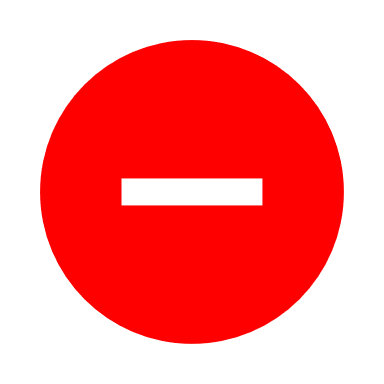 | **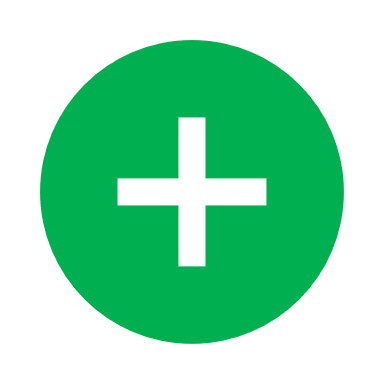** |
| **Sephton 2020** | Development only | 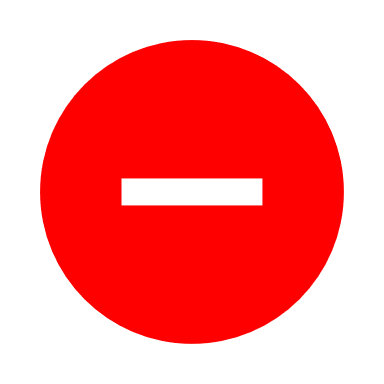 | 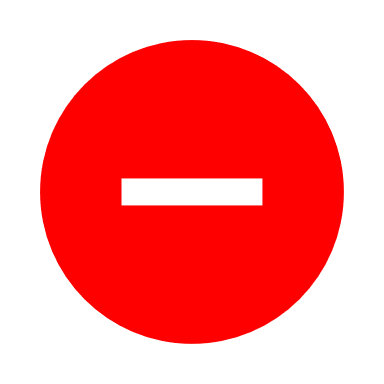 | 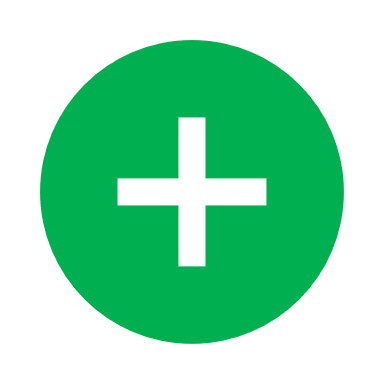 | 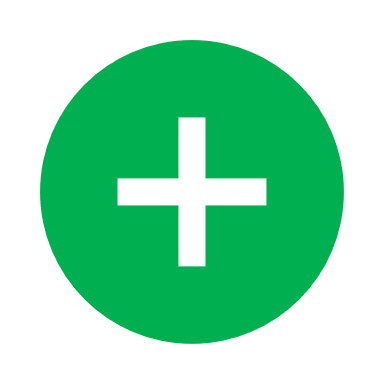 | 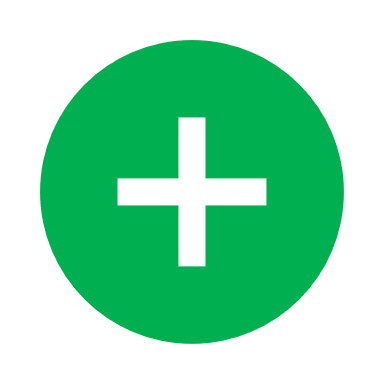 | 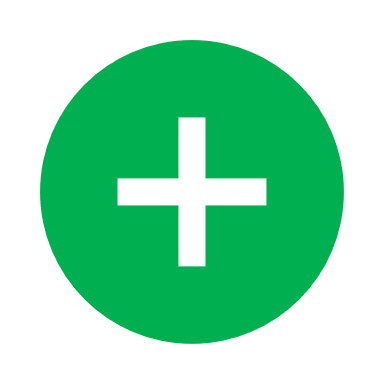 | 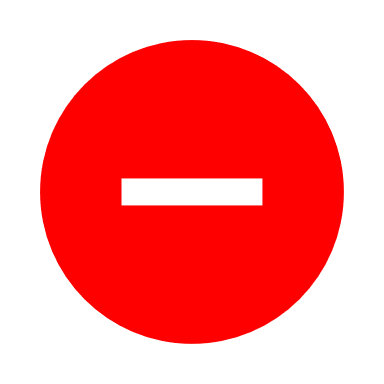 | 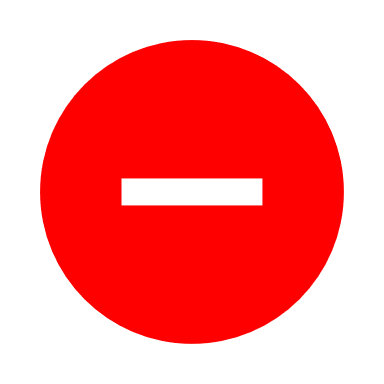 | **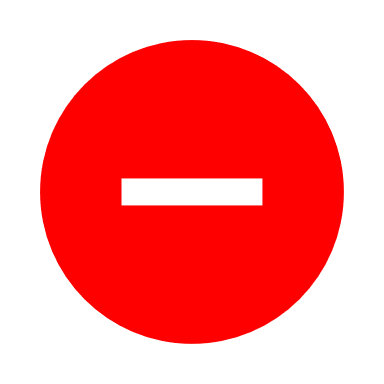** |
| **Ramkumar 2019** | Development and Validation | 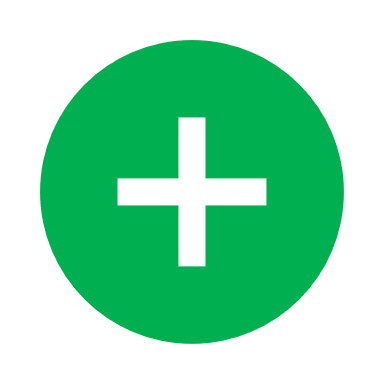 | 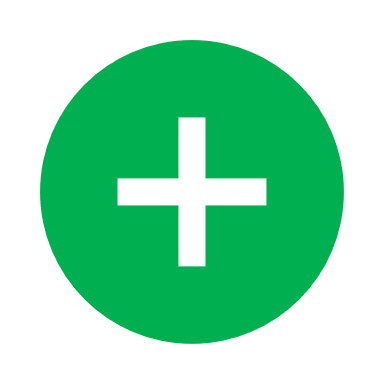 | 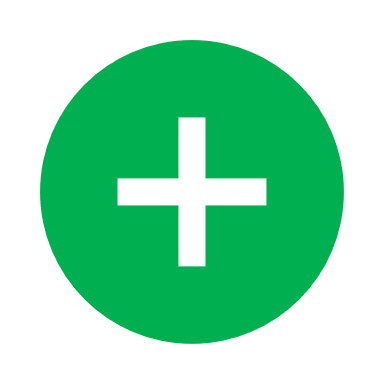 | 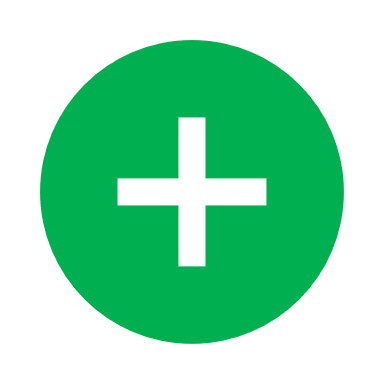 | 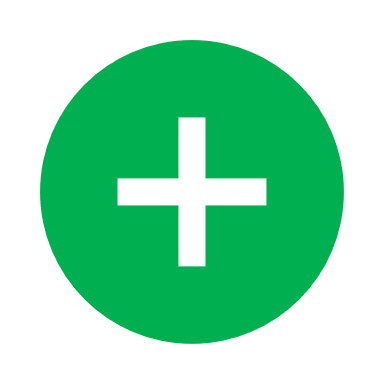 | 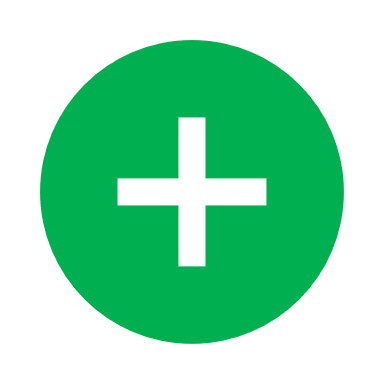 | 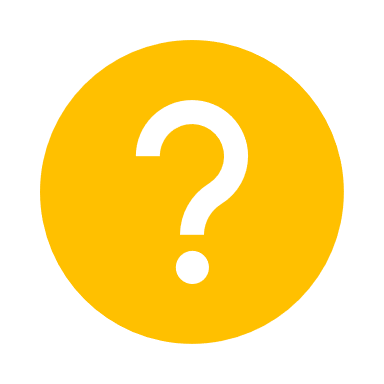 | 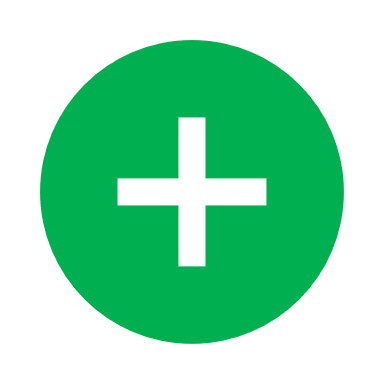 | **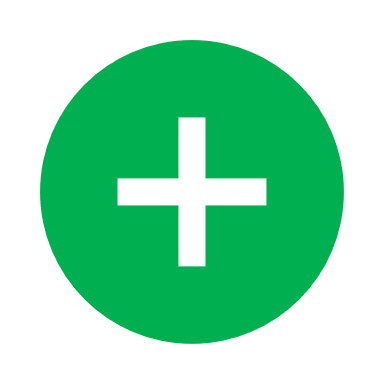** |
| **Winemaker 2015** | Development only | 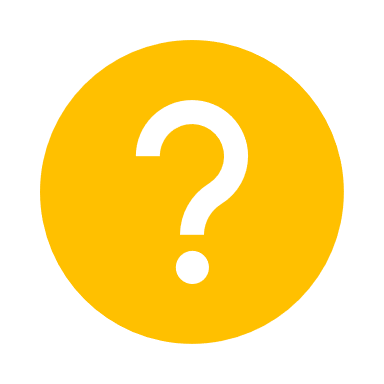 | 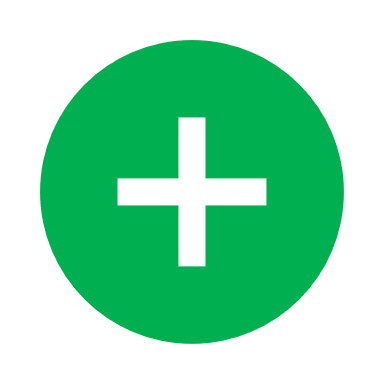 | 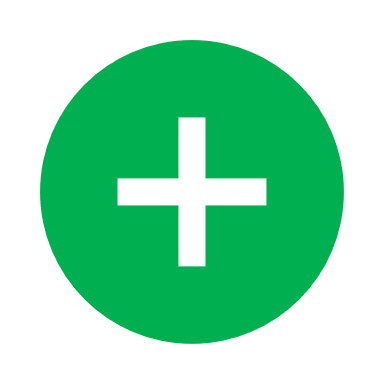 | 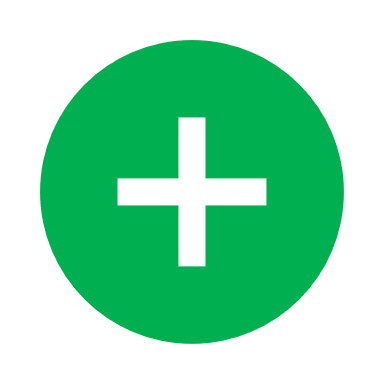 | 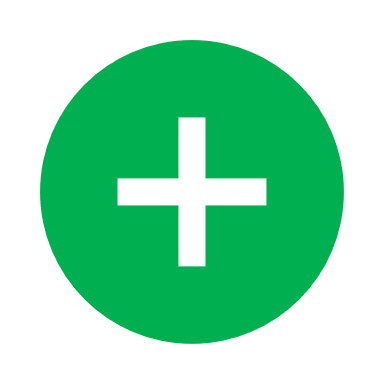 | 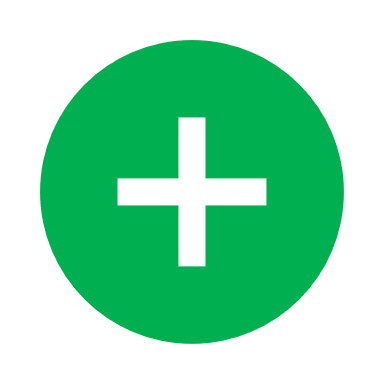 | 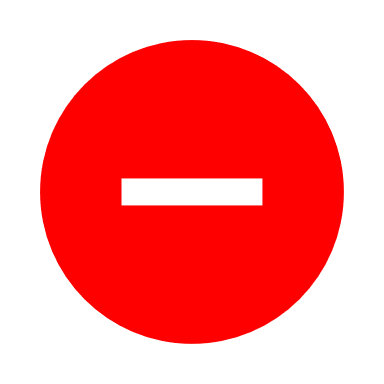 | 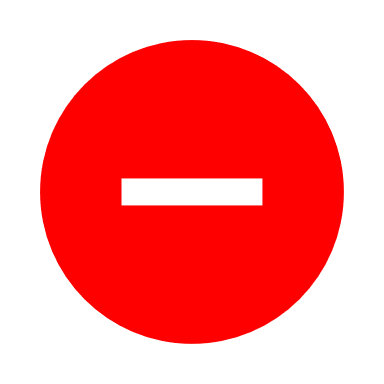 | **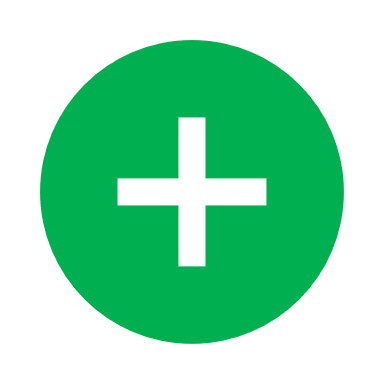** |

Table S8: Abbreviations:

| **Abbreviations** |  |
| --- | --- |
| LOS | Length of Stay |
| CCI | Charlson Comorbidity Index |
| FI | Frailty Index |
| ML | Machine Learning |
| AUROC | Area under Receiver Operating Curve |
| MSE | Mean Square Error |
| RMSE | Root Mean Square Error |
| TKA | Total Knee Arthroplasty |
| THA | Total Hip Arthroplasty |
| UKA | Unilateral Knee Arthroplasty |
| MEDPAR | Medicare or Centres for Medicare and Medicaid Services |
| CFS | Clinical Frailty Score |
| APR DRG | All Patient Refined |
| NRS 2002 | Nutritional Risk Score 2002 |
| MUST | Malnutrition Universal Screening Tool |
| SGA | Subjective Global Assessment |
| BMI | Body Mass Index |
| OME | OrthoMiDaS (Orthopedic Minimal Data Set) Episode of Care |
| NRI | Net Reclassification Index |
| PPV | Positive Predictive Value |
| NPV | Negative Predictive Value |
| AIC | Akaike’s Information Criterion |
| RSI | Risk Stratification Index |
| MAE | Mean Absolute Error |
| REML | Random-Effect Model Logistic |
|  |  |

### Table S9 Publication bias results for Egger’s test

**Regression-based Egger test for small-study effects**

Regression-based Egger test for small-study effects

Random-effects model

Method: REML

H0: beta1 = 0; no small-study effects

beta1 = 0.98

SE of beta1 = 5.562

z = 0.18

Prob > |z| = 0.8601

### Figure S1 : Frequency of predictor variables/features used in the retrieved General Surgery and Total Knee Arthroplasty LOS models

### Figure S2: Frequency of predictive metrics in included General Surgery and TKA models.


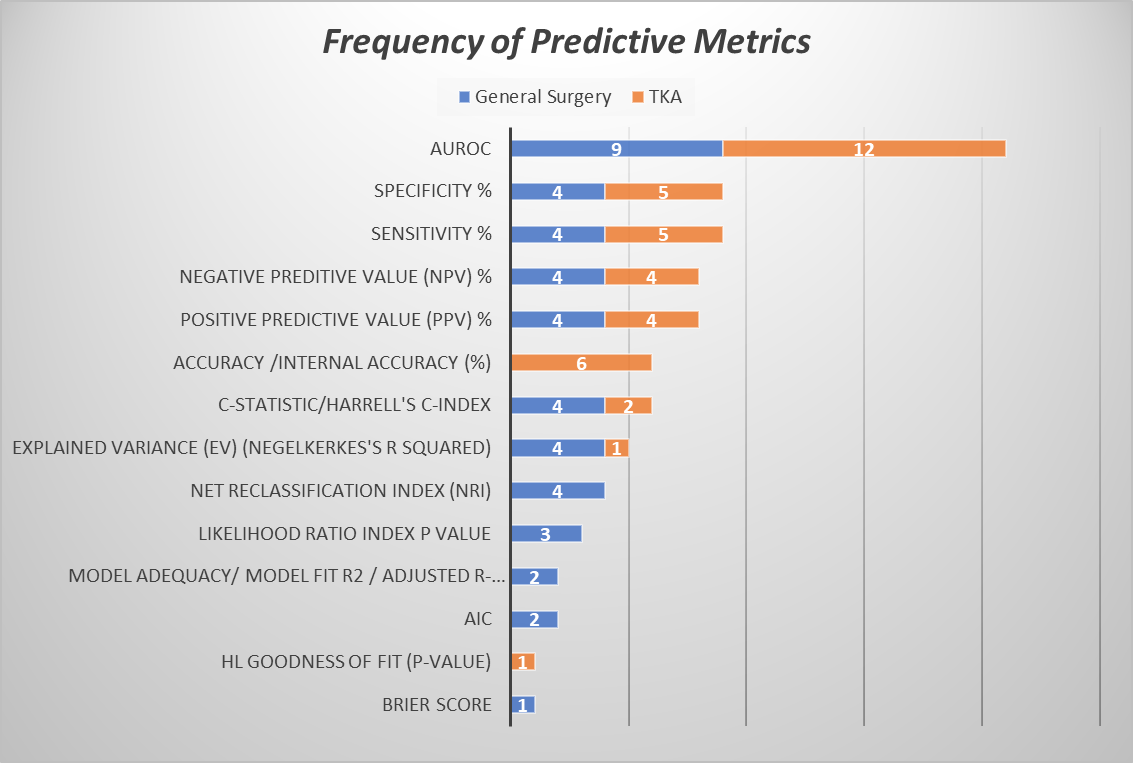


### Figure S3 Funnel plot
